# Fuelling repair: metabolic regulation of alveolar type 2 cell fate in lung regeneration and disease

**DOI:** 10.1242/dmm.052978

**Published:** 2026-09-30

**Authors:** Sophia Wugk, Sarah Kenny, Filza Masood, Ei Thant Htoo, Suzanne M. Cloonan

**Affiliations:** ^1^ School of Medicine, Trinity Biomedical Sciences Institute, Trinity College Dublin, Dublin D02 R590, Ireland; ^2^ Discipline of Clinical Medicine, Trinity Centre for Health Sciences, Tallaght University Hospital, Dublin D08 W9RT, Ireland; ^3^ Division of Pulmonary and Critical Care Medicine, Joan and Sanford I. Weill Department of Medicine, Weill Cornell Medicine, New York, NY 10065, USA

**Keywords:** Lung regeneration, Alveolar type 2 cell, Stem cell, Metabolism, Chronic lung disease, Interstitial lung disease, COPD, Pulmonary fibrosis

## Abstract

There is growing scientific interest in uncovering the mechanisms of lung regeneration, aiming to inform the development of innovative therapies for irreversible lung damage associated with chronic diseases. Cellular metabolism and its associated metabolites are increasingly recognised as active regulators of stem cell behaviour and tissue repair in other organs, yet little is known about how metabolic programmes and metabolite-mediated processes influence lung progenitor cell fate decisions. This Review explores current knowledge of how cellular metabolism directs the stem cell functions of alveolar type 2 (AT2) cells, the main epithelial progenitor cells of the distal lungs. Recent studies suggest that AT2 cell regenerative functions are linked to shifts in glycolytic and oxidative metabolism and require tight regulation of mitochondrial function and lipid metabolism for effective repair. Dysregulation of AT2 cell metabolism, as observed in chronic lung diseases, such as pulmonary fibrosis and chronic obstructive pulmonary disease, is emerging as a mechanism of regenerative failure and associated with respiratory disease progression. Improving our understanding of how AT2 cell metabolism ‘fails to fuel’ and starts ‘fuelling to fail’ lung repair is paramount for the development of innovative therapies that may slow or reverse the progression of chronic lung diseases.

## Teaser

**Summary:** Metabolic regulation is emerging as an active regulator of alveolar type 2 cell fate decisions in lung repair, with dysregulation of metabolic pathways contributing to impaired lung regeneration in chronic lung diseases.

## INTRODUCTION

The lung is essential for human health, enabling oxygen delivery through a vast alveolar surface specialised for efficient gas exchange. The human lung has some capacity for repair, allowing damaged areas to recover and restore functional tissue over time. However, this repair process is modest, and the lung cannot fully regenerate once substantial injury has occurred. Although the conducting airways, including the bronchi and bronchioles, possess greater regenerative capacity than the alveolar epithelium owing to the presence of multiple resident progenitor cell populations, it is the alveolar epithelium that represents the functionally critical site of gas exchange and is the primary area of regenerative failure in clinically significant lung disease (Basil et al., 2024). The alveolar epithelium consists of alveolar type 1 (AT1) cells, which form a thin barrier covering ∼95% of the alveolar surface to facilitate gas exchange, and alveolar type 2 (AT2) cells, which, despite comprising only ∼5% of the epithelium, are central to alveolar homeostasis and repair (Fig. 1) (Payne and Wellikof, 2012). AT2 cells are highly metabolically active and support surfactant synthesis, secretion and recycling, while also serving as the resident stem cells of the alveolar epithelium with the capacity for self-renewal and differentiation into AT1 cells following injury (Rooney et al., 1994; Barkauskas et al., 2013; Desai et al., 2014). Such alveolar regeneration occurs through a continuum of transitional alveolar differentiation intermediate (ADI) states (also known as transitional cell states) that bridge activated AT2 cells to terminally differentiated AT1 cells. Following injury to the alveolar epithelium, surfactant protein C (*SFTPC*)^+^ AT2 cells first enter an early keratin 8 (*KRT8*)^+^/claudin 4 (*CLDN4*)^+^ transitional state before progressing to a later galectin 3 (*LGALS3*)^+^ transitional state that gradually acquires AT1 characteristics, and they ultimately differentiate into mature AT1 cells expressing advanced glycosylation end-product specific receptor (*AGER*), podoplanin (*PDPN*) and homeodomain-only protein homeobox (*HOPX*) (Strunz et al., 2020; Kobayashi et al., 2020; Choi et al., 2020; Shen et al., 2022). Disruption of progression through these transitional states is increasingly recognised as a central mechanism underlying failed alveolar repair in chronic lung disease (Lin et al., 2022; Wang and Tang, 2020; Sanches Santos Rizzo Zuttion et al., 2022).

Emerging evidence indicates that progression through these regenerative cell states is also tightly coupled to dynamic metabolic reprogramming, suggesting that the regenerative capacity of AT2 cells depends on metabolic plasticity (Li et al., 2020; Choi et al., 2020). Although individual metabolic reactions occur over relatively short timescales, sustained metabolic programmes have been shown to establish durable cellular states through coordinated metabolic, transcriptional and epigenetic remodelling, influencing biological processes over days, months and, in some cases, years (Ren et al., 2017; Arts et al., 2016; Carey et al., 2015; Oburoglu et al., 2014; Netea et al., 2016). In this Review, we summarise current understanding of metabolic reprogramming during AT2 cell-mediated lung repair. Specifically, we examine how dynamic regulation of glycolysis, fatty acid oxidation (FAO) and oxidative phosphorylation (OXPHOS) supports distinct stages of alveolar repair, and highlight the contributions of lipid, amino acid and micronutrient metabolism to epithelial regeneration. We also discuss current limitations of the field, including the fact that many mechanistic insights are inferred from perturbation of metabolic enzymes and pathways rather than direct manipulation of individual metabolites, as well as explore how metabolic dysfunction contributes to failed repair and chronic lung disease.

## Metabolic reprogramming in lung regeneration

Alveolar regeneration is a complex and energetically demanding process in which AT2 cells must activate, proliferate, transition through intermediate states and, ultimately, differentiate into mature AT1 cells to restore alveolar integrity. Increasing evidence from stem cell biology suggests that metabolism is not merely a passive supplier of energy and biosynthetic substrates for such processes but actively regulates cell fate decisions by affecting signalling pathways, redox balance and epigenetic state (Kwak et al., 2024; Chaves-Perez et al., 2025; Andersson et al., 2025; Lalioti et al., 2025). Emerging studies now indicate that progression through the AT2-to-AT1 differentiation continuum is accompanied by distinct metabolic transitions, raising the possibility that metabolic reprogramming is also a fundamental regulator of alveolar regeneration.

**Fig. 1.**
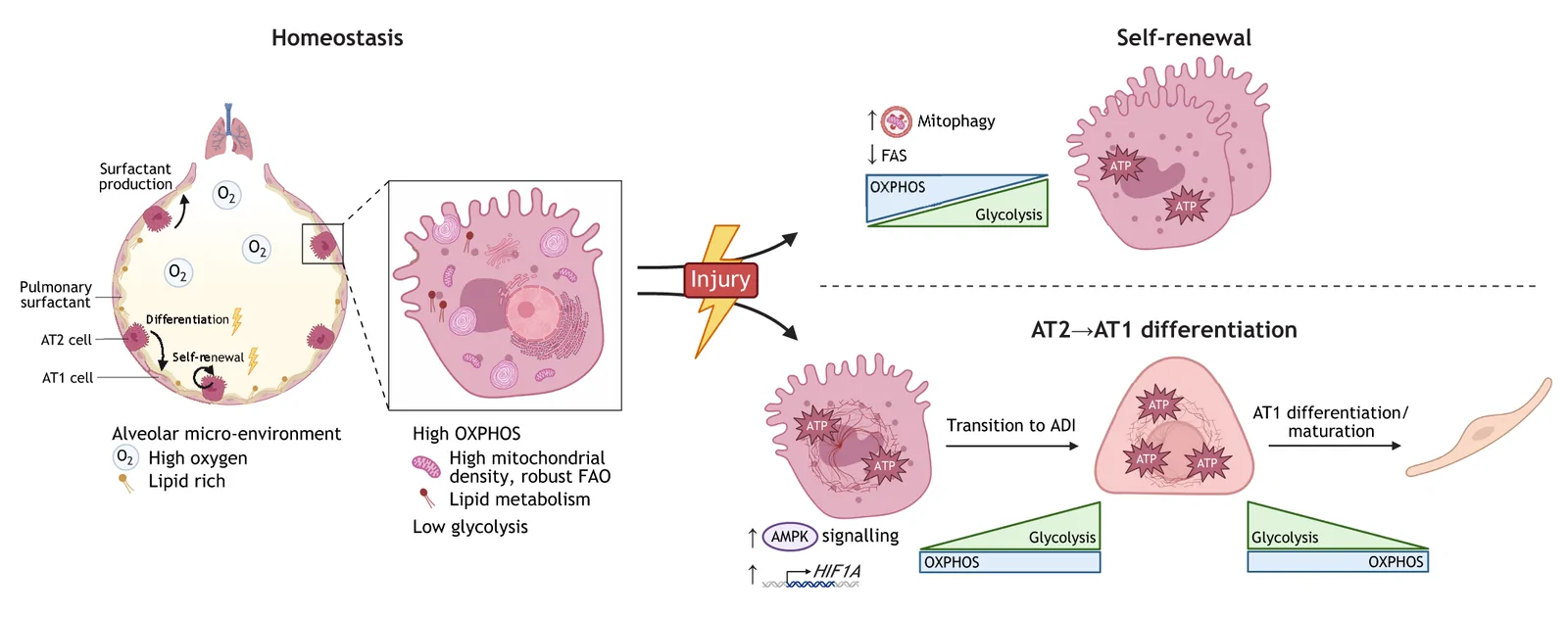
**Schematic representation of AT2 cell metabolism in alveolar regeneration.** AT2 cells predominantly utilise OXPHOS for energy production in homeostasis, with high oxygen tension driving this metabolic phenotype. Upon injury, AT2 cells upregulate glycolysis to meet the energetic demands of self-renewal and differentiation. AT2-to-AT1 differentiation specifically requires AMPK-induced glycolytic metabolism to enter the transitional ADI cell state, followed by subsequent inhibition and a shift toward mitochondrial respiration for terminal differentiation. AMPK, AMP-activated protein kinase; ADI, alveolar differentiation intermediate cell; ATP, adenosine triphosphate; AT1, alveolar type 1; AT2, alveolar type 2; FAO, fatty acid oxidation; FAS, fatty acid synthesis; *HIF1A*, hypoxia inducible factor 1, alpha subunit; OXPHOS, oxidative phosphorylation. Created in BioRender by Cloonan, S. (2026). https://BioRender.com/ahl62fb. This figure was sublicensed under CC-BY 4.0 terms.

Under homeostatic conditions, AT2 cells predominantly rely on OXPHOS and FAO to sustain the enhanced metabolic demand of their specialised cell functions, such as surfactant production (Wheaton and Chandel, 2011; Semenza, 2012; Lottes et al., 2014; Cui et al., 2019). Consistent with this, AT2 cells exhibit a high mitochondrial density (Massaro et al., 1975), and depend on intact mitochondrial function for survival and maintenance (Wang et al., 2023; Han et al., 2023). However, following injury, this metabolic dependence changes, with activated AT2 cells undergoing dynamic metabolic reprogramming that appears to parallel their progression through distinct regenerative cell states. Emerging evidence suggests that entry into the early *KRT8*^+^/*CLDN4*^+^ transitional state is associated with increased glycolytic activity, whereas successful terminal differentiation into mature AT1 cells requires restoration of mitochondrial respiration and oxidative metabolism (Fig. 1) (Choi et al., 2020; Li et al., 2023; Wang et al., 2023; Han et al., 2023). In addition, emerging evidence indicates that lipid, amino acid, micronutrient and energy-sensing pathways contribute to the regulation of distinct stages of alveolar regeneration, underscoring the complexity of metabolic control over AT2 cell fate decisions. The following sections discuss how these interconnected metabolic programmes influence AT2 cell activation, proliferation, transitional-state progression and differentiation, and how their dysregulation contributes to failed repair in chronic lung disease.

### Glycolysis and OXPHOS metabolism

Glycolysis and OXPHOS are the two main pathways that cells use to generate adenosine triphosphate (ATP). Glycolysis, occurring in the cytoplasm, breaks down glucose into pyruvate, rapidly producing a small amount of ATP (often referred to as GlycoATP), without requiring oxygen. By contrast, OXPHOS takes place in mitochondria, in which electrons from nicotinamide adenine dinucleotide (NADH) and flavin adenine dinucleotide (FADH_2_) drive the electron transport chain (ETC) to produce large amounts of ATP (MitoATP) in an oxygen-dependent process. The balance between these pathways regulates cellular energy availability during homeostasis and upon injury.

AT2 cell self-renewal and differentiation are both highly energy-intensive processes that require significant amounts of ATP to facilitate DNA replication, cell-cycle progression and biosynthesis of macromolecules required for cell division, or for the cytoskeletal remodelling from cuboidal AT2 cells to flattened AT1 cells during differentiation (Wang et al., 2023). To meet the high energy demand of regeneration, AT2 cells significantly upregulate their total ATP production primarily through glycolysis in murine models of bleomycin-induced lung injury and fibrosis or during *ex vivo* differentiation of primary murine AT2 cells (Wang et al., 2023; Li et al., 2020), as glycolysis produces ATP at a faster rate than oxidative respiration (Pfeiffer et al., 2001; Lunt and Vander Heiden, 2011). Supporting this, conditional genetic deletion of *Glut1* (also known as *Slc2a1*), encoding a major glucose transporter required for glucose uptake, in murine AT2 cells significantly impaired AT2 cell proliferation and organoid-forming capacity following bleomycin injury (Li et al., 2020). Additionally, the pentose phosphate pathway, which is fuelled by intermediates from glycolysis, was upregulated in regenerating AT2 cells in murine bleomycin injury models to support nucleotide biosynthesis. Pharmacological inhibition of this pathway also reduced primary murine AT2 cell proliferation and organoid formation *ex vivo* (Li et al., 2020). In mice, AT2 cell transition in response to bleomycin injury is also associated with increased rates of glucose uptake and glycolytic output, with early-stage transitional (*Krt8*^+^ *Cldn4*^+^) cells showing increased expression of glycolytic genes including hypoxia inducible factor 1, alpha subunit (*Hif1a*), a key regulator of glycolysis (Semenza, 2012), as well as increased HIF1α-dependent glycolytic metabolism. However, persistent glycolytic signalling impaired their progression to mature AT1 cells, suggesting that downregulation of glycolysis is required for terminal differentiation (Choi et al., 2020).

In contrast to glycolysis, evidence for OXPHOS regulation has emerged primarily from studies examining AT2 cell differentiation rather than proliferation, with some evidence that murine AT2 cells entering the early transitional cell state do not alter mitochondrial ATP production (Wang et al., 2023). However, transcriptomic analyses of bleomycin injury-induced alveolar regeneration identified enrichment of OXPHOS-associated gene signatures within *Krt8*^+^ transitional cells, suggesting increased engagement of mitochondrial metabolism during progression through the differentiation programme (Strunz et al., 2020). Consistent with this, disruption of mitochondrial electron transport by genetic deletion of the ETC complex I subunit-encoding NADH:ubiquinone oxidoreductase core subunit S2 (*Ndufs2*) in murine AT2 cells resulted in accumulation of *Krt8*^+^ transitional cells and impaired terminal differentiation into mature AT1 cells during postnatal lung development (Han et al., 2023). This suggests a requirement for functional OXPHOS for terminal AT2 cell differentiation to AT1 cells. In line with this, a subset of transitioning murine AT2 cells expressing AT1 markers demonstrated increased expression of genes involved in OXPHOS during alveolar repair after bleomycin injury, linking restoration of oxidative metabolism with successful acquisition of AT1 identity (Li et al., 2023). Furthermore, loss of mitochondrial pyruvate carrier 2 (*Mpc2*), a key gatekeeper of mitochondrial pyruvate import that fuels the tricarboxylic acid cycle (TCA) cycle and OXPHOS, impaired AT2 cell differentiation into both *Krt8*^+^ transitional and mature AT1 cells in murine bleomycin injury models (He et al., 2026). Collectively, these findings suggest that, although glycolytic metabolism supports early regenerative responses, intact mitochondrial respiration and OXPHOS are required for efficient progression through transitional states to terminal AT1 cell differentiation.

Lastly, mitochondrial shape and size are continuously regulated by fission and fusion processes to facilitate metabolic adaptation (Chung et al., 2019; Mishra et al., 2014; Toyama et al., 2016), which play an important role in stemness and hematopoietic and other stem cell fate decisions by regulating OXPHOS activity (Papa et al., 2019; Prieto et al., 2016; Maryanovich et al., 2015; Khacho et al., 2016). Although mitochondrial fusion is not essential for AT2 cell proliferation, AT2-specific deletion of the mitochondrial fusion protein-encoding genes *Mfn1* and *Mfn2* in mice disrupted surfactant lipid metabolism, impaired phospholipid and cholesterol synthesis, and promoted spontaneous as well as bleomycin-induced lung fibrosis, highlighting an important role for mitochondrial fusion in maintaining AT2 cell function and regenerative capacity (Chung et al., 2019). Studies using hematopoietic and embryonic stem cells have shown that, to facilitate differentiation, elevated mitochondrial biogenesis increases mitochondrial mass and membrane potential to enhance cellular OXPHOS activity (Ho et al., 2017; Cho et al., 2006; Mohrin et al., 2018); however, little is known about this process in the alveolar epithelium.

### Lipid metabolism

Under homeostatic conditions, AT2 cells maintain highly efficient lipid metabolism to support surfactant synthesis and recycling, performing robust fatty acid synthesis (FAS) (Das, 1980; Maniscalco et al., 1989; Patterson et al., 1988), as well as FAO, inside their mitochondria to generate high levels of acetyl-CoA, NADH and FADH_2_ to fuel the TCA cycle and, subsequently, OXPHOS (Cui et al., 2019). AT2 cell lipid metabolism is further supported by neighbouring lipofibroblasts, which act as a local lipid reservoir and provide metabolic substrates essential for surfactant production and AT2 cell maintenance (Schultz et al., 2002; Besnard et al., 2009). Effective lipid metabolism is, therefore, a key regulator of AT2 cell-mediated repair after injury. Partial deletion of ABCA3, a transport protein importing cytoplasmic phospholipids into lamellar bodies, leads to impaired surfactant homeostasis alongside alveolar injury and inflammation in mice, while rescue of ABCA3 expression restores alveolar repair through enhanced AT2 cell proliferation (Rindler et al., 2017). These findings suggest that maintenance of surfactant lipid homeostasis is not only required for alveolar function but also for preserving the regenerative competence of AT2 cells.

Following bleomycin injury in mice, activated AT2 cells induce autophagy to suppress FAS, and redirect metabolic resources towards enhanced glucose utilisation to support rapid proliferation and lung regeneration (Li et al., 2020). Consistent with this, pharmacological inhibition of fatty acid synthase (FASN), the rate-limiting enzyme for *de novo* fatty acid synthesis, enhanced the proliferation and organoid-forming capacity of primary murine AT2 cells isolated following bleomycin injury, suggesting that suppression of FAS promotes the regenerative expansion of AT2 cells during alveolar repair (Li et al., 2020). Supporting this concept, overall lipid deficiency impairs AT2 cell regenerative functions, while supplementation with a fatty acid-rich lipid mixture restored progenitor renewal capacity and promoted AT2 cell proliferation and differentiation in both human idiopathic pulmonary fibrosis (IPF)-derived and murine bleomycin injury-derived alveolar epithelial progenitor cultures (Liang et al., 2024). However, chronic AT2 cell-specific deletion of *Fasn* in mice disrupted surfactant lipid homeostasis and exacerbated cigarette smoke (CS)-induced emphysema, suggesting that, although transient suppression of FAS supports regenerative proliferation, sustained disruption of lipid synthesis impairs long-term AT2 cell function and alveolar integrity (Fan et al., 2023).

In contrast to the beneficial effects of transient suppression of FAS during proliferation, FAO appears to remain essential for AT2 cell function and lung repair (Chung et al., 2024). Recent studies demonstrated that AT2 cell-specific deletion of carnitine palmitoyltransferase 1a, liver (*Cpt1a*), encoding the rate-limiting enzyme of mitochondrial long-chain FAO, altered surfactant phospholipid homeostasis and inflammatory signalling without significantly reducing ATP production in mice, highlighting a broader role for FAO in regulating AT2 cell biology beyond bioenergetics (Chung et al., 2024). Impaired FAO also contributes to aberrant lung repair and promotes the development of acute lung injury and fibrosis in murine models, including LPS-induced injury, SFTPC I73T mutation and bleomycin challenge (Angeles-Lopez et al., 2025; Cui et al., 2019; Rodríguez et al., 2025), providing evidence that intact FAO is required to maintain AT2 cell regenerative potential. Given the emerging requirement for OXPHOS during terminal differentiation, FAO may provide an important source of mitochondrial substrates supporting AT2-to-AT1 differentiation. In line with this theory, genetic depletion of *Cpt1a* to inhibit FAO in mice induced accumulation of *Krt8*^+^ transitional cells displaying impaired mitochondrial function and promoted susceptibility to fibrosis following bleomycin challenge (Angeles-Lopez et al., 2025), providing direct evidence that FAO is required for progression beyond the transitional cell state. Similarly, airway epithelial progenitor (basal) cells exhibit a metabolic shift away from glycolysis toward FAO during differentiation, and disruption of FAO impaired epithelial maturation in both primary murine airway organoid cultures and naphthalene-induced airway repair models (Crotta et al., 2023). Importantly, no studies have directly characterised FAS or FAO activity across defined *Krt8*^+^ transitional states. Given the requirement for glycolysis during early transitional-state entry and the apparent requirement for OXPHOS during terminal differentiation, it is tempting to speculate that transitional AT2 cells undergo a coordinated metabolic switch characterised by suppression of FAS and restoration of FAO to support OXPHOS-dependent differentiation. However, direct evidence for this model is currently lacking.

Collectively, these findings indicate that tight and dynamic regulation of lipid metabolism is central to AT2 cell regenerative behaviour, highlighting that proliferating AT2 cells transiently suppress FAS, whereas maintenance and subsequent restoration of oxidative lipid metabolism may be required for progression through transitional states and terminal differentiation during alveolar repair. However, the exact mechanisms of how lipid metabolism regulates AT2 cell regenerative functions remain unclear. Future studies are needed to determine how individual lipid species influence metabolic signalling and whether dynamic shifts between FAS and FAO regulate progression through transitional cell states and terminal AT1 cell differentiation.

### Amino acid metabolism

Amino acids are fundamental metabolic substrates that support cellular bioenergetics, redox balance and biosynthetic pathways, thereby integrating nutrient availability with cell growth, differentiation and stress responses. As such, amino acids have been implicated in regulating stem cell fate decisions (Shyh-Chang et al., 2013; Shiraki et al., 2014). Although amino acid metabolism in stem cell activity has been discussed in previous literature reviews (Zeng et al., 2025; Liu et al., 2019), literature on amino acid metabolism in lung stem cell populations, specifically AT2 cells, largely remains limited to a few studies on glutamine metabolism (Wang et al., 2022b; Sappington et al., 2016).

Glutamine is a critical metabolite supporting mitochondrial respiration and ATP production. It is transported into cells and converted to glutamate by mitochondrial glutaminase-1 (GLS1; also known as GLS), after which glutamate is further metabolised to α-ketoglutarate via glutamic-pyruvate transaminase-2 (GPT2) or glutamic-oxaloacetic transaminase-2. α-Ketoglutarate subsequently enters the TCA cycle, thereby enhancing ATP production. In parallel, glutamine serves as a major nitrogen donor for the synthesis of nonessential amino acids, nucleotides and the antioxidant glutathione. Consequently, glutamine metabolism is essential for the self-renewal and differentiation of various stem cell types (Tohyama et al., 2016; Oburoglu et al., 2014; Carey et al., 2015), and pharmacological inhibition of key enzymes involved in glutamine metabolism – GLS1 and GPT2 – markedly reduced primary murine AT2 cell proliferation and differentiation into AT1 cells in *ex vivo* alveolar organoid cultures (Wang et al., 2022b; Sappington et al., 2016)*.*

Although glutamine metabolism represents the most-studied amino acid pathway in AT2 cells, emerging evidence suggests that transport of other amino acids may also regulate AT2 stem cell function. For example, a recent study identified the neutral amino acid transporter LAT1 (encoded by *SLC7A5*), which mediates uptake of large neutral amino acids including leucine, as a regulator of AT2 cell proliferation (Fujimoto et al., 2026; Salisbury and Arthur, 2018). Specifically, LAT1 was identified as a functional cargo within lipofibroblast-derived extracellular vesicles, and knockdown of LAT1 impaired the growth of primary murine AT2 cell-derived organoids, whereas supplementation with LAT1-enriched vesicles enhanced organoid growth (Fujimoto et al., 2026). These findings suggest that amino acid availability, in addition to amino acid metabolism itself, may contribute to regulating AT2 cell regenerative behaviour.

Overall, research explicitly focusing on amino acid metabolism in lung stem cells is still developing, and our understanding of many metabolic processes, including specific mechanisms of how amino acids actively regulate AT2 stem cell activity, remains incomplete.

### Essential micronutrients and cofactors

Along with major metabolites, micronutrients and metabolic cofactors are essential for regulating cellular metabolism. Several vitamins are central to metabolic processes supporting cellular proliferation and differentiation. Vitamin A-derived retinoids are important for the cell cycle and proliferation, and supplementation with retinoic acid promotes the proliferation of foetal AT2 cells through enhanced S-phase entry and augments differentiation into AT1 cells (Gao et al., 2015). Importantly, the effects of retinoic acid appear to be highly dependent on the timing of administration. In murine models, retinoic acid pre-treatment prior to alveolar injury enhanced regenerative responses (Gokey et al., 2021), whereas administration to mice following elastase-induced alveolar injury failed to promote, and in some contexts impaired, alveolar regeneration (Stolk et al., 2012). This suggests that the effects of retinoic acid are highly dependent on the temporal phases during injury and regeneration.

In addition, B-group vitamins including vitamin B9 (folate) and B12 support proliferative metabolism by fuelling one-carbon metabolism required for nucleotide synthesis (Lyon et al., 2020; Yee et al., 2022). Vitamin B3 serves as a precursor for nicotinamide adenine dinucleotide hydrate (NAD^+^), a central cofactor that supports cellular metabolism through its redox activity in glycolysis, FAO and the TCA cycle (Covarrubias et al., 2021). NAD^+^ levels have been implicated as regulators of stem cell proliferation and differentiation (Navas and Carnero, 2021; Lamprecht et al., 2026), and supplementing primary murine AT2 cells *ex vivo* with nicotinamide mononucleotides ameliorated senescence in passaged cells compared to untreated control cells (Fang et al., 2021). Similarly, administering nicotinamide mononucleotides to 8- to 10-month-old mice alleviated AT2 cell senescence in an *in vivo* bleomycin-induced murine model of fibrosis (Fang et al., 2021).

Futhermore, vitamin C supports primary guinea pig AT2 cell resistance to oxidative injury (Brown et al., 1997). Although further AT2 cell-focused studies are lacking, vitamin C supplementation limited oxidative stress and mitochondrial dysfunction in an *in vitro* model of chronic obstructive pulmonary disease (COPD) consisting of particulate matter (PM)2.5-treated bronchial epithelial (Beas2B) cells and in the lungs of PM2.5-exposed mice (Bai et al., 2025). Whether vitamin C supports AT2 cell regenerative functions by maintaining mitochondrial integrity necessary for terminal differentiation remains to be determined. Together, these findings highlight the role of micronutrients in stem cell functions and implicate vitamin deficiencies in impaired AT2-cell mediated lung regeneration.

Finally, trace elements – such as iron, zinc and copper – support fundamental enzymatic processes of cellular metabolism. Iron, for example, functions as an essential cofactor for many eukaryotic proteins, such as iron–sulphur cluster proteins, which are essential for mediating electron transfer in metabolic respiration (Read et al., 2021), as well as DNA replication, repair and cell-cycle progression (Zhang, 2014). As such, functional iron insufficiency is associated with impaired potential for AT2 cell proliferation and differentiation (Zhuang et al., 2025). By contrast, iron overload can lead to oxidative stress, culminating in senescence or ferroptosis of AT2 cells (Maus et al., 2023; Cheng et al., 2021). Tight regulation of iron availability, providing sufficient iron for proper cell function while limiting iron overload, is thus essential for effective AT2 cell-mediated lung repair. Furthermore, effective zinc metabolism is required for AT2 cell regenerative functions. Specifically, genetic depletion of *Zip8* (also known as *Slc39a8*), encoding a zinc transporter that can also transport iron (Wang et al., 2012), in AT2 cells led to the development of spontaneous lung fibrosis in mice and impaired AT2 cell self-renewal and differentiation capacity in an *ex vivo* AT2 cell-derived organoid model (Liang et al., 2022). Supplementation with exogenous zinc restored AT2 cell regenerative functions and improved the restorative response to bleomycin injury in mice (Liang et al., 2022). Literature evaluating the role of copper metabolism in AT2 cell metabolism is sparse. A recent study has proposed that cuproptosis, a copper-dependent programmed cell death, of bronchial epithelial cells contributes to airway remodelling in a chronic murine model of asthma (Wang et al., 2026). However, whether copper metabolism directly contributes to AT2 cell regenerative functions remains to be determined. Overall, how trace metal metabolism regulates AT2 cell biology, particularly regenerative functions, remains largely unexplored.

### Energy-sensing pathways

As metabolism evidently plays a central role in lung regeneration, energy-sensing pathways drive AT2 cell fate decisions by regulating shifts in cellular metabolism. AT2 cells respond to the increased energetic demands of cytoskeletal remodelling during lung repair primarily by activation of the adenosine monophosphate (AMP)-activated protein kinase (AMPK) pathway, which drives a shift toward preferential GlycoATP generation. AMPK is a critical regulator of cellular energy homeostasis and functions as an energy sensor, as low energy levels marked by an increased AMP:ATP ratio activate AMPK signalling (Wang et al., 2023; Hardie et al., 2012). Under homeostatic conditions, AT2 cells express minimal activated/phosphorylated AMPK (p-AMPK), but, following lung injury, p-AMPK levels are significantly upregulated, indicating activation of the energy-sensing pathway during regeneration (Wang et al., 2023). Specifically, pharmacological inhibition of the AMPK-regulated glycolytic enzyme 6-phosphofructo-2-kinase/fructose-2,6-bisphosphatase 2 (PFKFB2) in primary murine AT2 cells and organoid models reduced glycolytic ATP production and impaired AT2-to-AT1 differentiation owing to inadequate ATP availability for the cytoskeletal remodelling required during regeneration (Wang et al., 2023).

Energy sensing in AT2 cells also involves the mammalian target of rapamycin (mTOR) signalling pathway, which serves as a key regulator of cell metabolism, growth, proliferation and survival by integrating a variety of intracellular and extracellular signals (Laplante and Sabatini, 2009). mTOR activity generally promotes cell proliferation and biosynthesis when nutrients are abundant, while suppression of mTOR during metabolic stress induces autophagy to sustain catabolic metabolism (Laplante and Sabatini, 2009; Meng et al., 2018). As such, mTOR signalling plays a role in regulating stem cell functions (Luo et al., 2014; Chen et al., 2008). In the lung, persistent activation of mTOR signalling has been implicated in bleomycin-induced fibrosis, at least in part through suppression of autophagy and disruption of normal epithelial repair processes (Gui et al., 2015). Inhibition of autophagy impairs murine AT2 cell proliferation following bleomycin injury (Li et al., 2020), highlighting the importance of maintaining catabolic metabolic programmes during regeneration. Impaired autophagy also promotes AT2 cell senescence and defective repair. In a murine carbon nanotube injury model, restoration of autophagic flux reduced epithelial senescence and subsequent fibrotic remodelling in the lung (Zhang et al., 2023). Similarly, in human lymphangioleiomyomatosis (LAM) samples, AT2 organoid co-culture systems and murine LAM models, tuberin (TSC2)-dependent mTOR activation induced IL-6-mediated AT2 cell senescence and impaired epithelial wound repair, effects that were partially reversed by mTOR inhibition (Babaei-Jadidi et al., 2025).

Overall, energy-sensing pathways, including AMPK and mTOR signalling, function as central metabolic checkpoints that coordinate energy availability with AT2 cell proliferation, differentiation, autophagy and senescence. Dysregulation of these pathways, therefore, has the potential to impair alveolar regeneration and promote chronic lung disease.

### Autophagy and reactive oxygen species

Reactive oxygen species (ROS) are a byproduct of OXPHOS, and AT2 cells naturally produce ROS through their high OXPHOS activity (Lottes et al., 2014). Owing to their oxygen-rich environment, however, AT2 cells are exceptionally susceptible to oxidative stress, causing oxidative damage to lipids, proteins and nucleic acids, impairing proper cell function, and promoting senescence and cell death (Harijith et al., 2022; Messier et al., 2013; Sun et al., 2024). In murine bleomycin models, increased ROS levels activate autophagy as a protective mechanism to limit excess ROS production and oxidative stress-induced epithelial damage (Li et al., 2020). This switches AT2 cell metabolism towards glycolysis, in turn promoting the proliferation of surviving AT2 cells after injury (Li et al., 2020; Sun et al., 2024). Conversely, AT2-specific autophagy deficiency impaired proliferation in primary murine AT2 cells and reduced their organoid-forming capacity (Li et al., 2020), highlighting the importance of autophagy for lung regeneration after injury. Interestingly, this suggests that chronically elevated ROS levels impair AT2 cell differentiation into mature AT1 cells, as the final stages of differentiation require inhibition of glycolysis and a shift toward OXPHOS.

In line with this, efficient mitophagy is critical for mitochondrial quality control, selectively eliminating dysfunctional or damaged mitochondria, thereby limiting pathological ROS accumulation and preserving AT2 cell regenerative functions. Supporting this, genetic depletion or pharmacological inhibition of ubiquitin specific peptidase 30 (*Usp30*), a negative regulator of mitophagy, reduced disease severity in murine bleomycin-induced fibrosis and influenza pneumonia injury models (Wu et al., 2026). Furthermore, *Usp30* depletion enhanced the proliferation and differentiation of primary murine AT2 cells in organoid cultures, suggesting that mitophagy supports AT2 cell regenerative function (Wu et al., 2026).

Beyond the activation of autophagy, ROS can act as signalling molecules regulating stem cell fate decisions (Ludin et al., 2014; Jang and Sharkis, 2007; Ito et al., 2004). Interestingly, rather than absolute ROS levels, the flux from low to moderate levels specifically regulates the self-renewal and proliferation of human and mouse airway basal stem cells by activating the nuclear factor erythroid 2-related factor 2 (NRF2)–Notch signalling pathway (Paul et al., 2014). Notch signalling is clearly established in the literature to control AT2 cell differentiation, with sustained elevation of Notch signalling impairing their differentiation to mature AT1 cells (Wang et al., 2024). This link further implicates ROS as a regulator of AT2 cell differentiation. The specific role of ROS as signalling molecules regulating AT2 cell regenerative functions has not yet been characterised. However, chronically elevated ROS, as observed in IPF lungs, impairs AT2 cell proliferation and promotes cellular senescence (Li et al., 2020; Lehmann et al., 2017; Cui et al., 2011).

## Dysregulated AT2 cell metabolism in ageing and chronic lung diseases

Aberrant AT2 cell metabolism has been implicated in many chronic lung diseases, in part associated with failure in the regenerative capacity of AT2 cells (Fig. 2, Table 1). Therefore, it is imperative to better understand how, when and why this metabolism ‘fails to fuel’ and starts ‘fuelling to fail’ to inform the development of potential AT2 cell-directed therapies that not only combat the progression of chronic lung diseases but, preferably, also reverse lung epithelial damage.

**Fig. 2.**
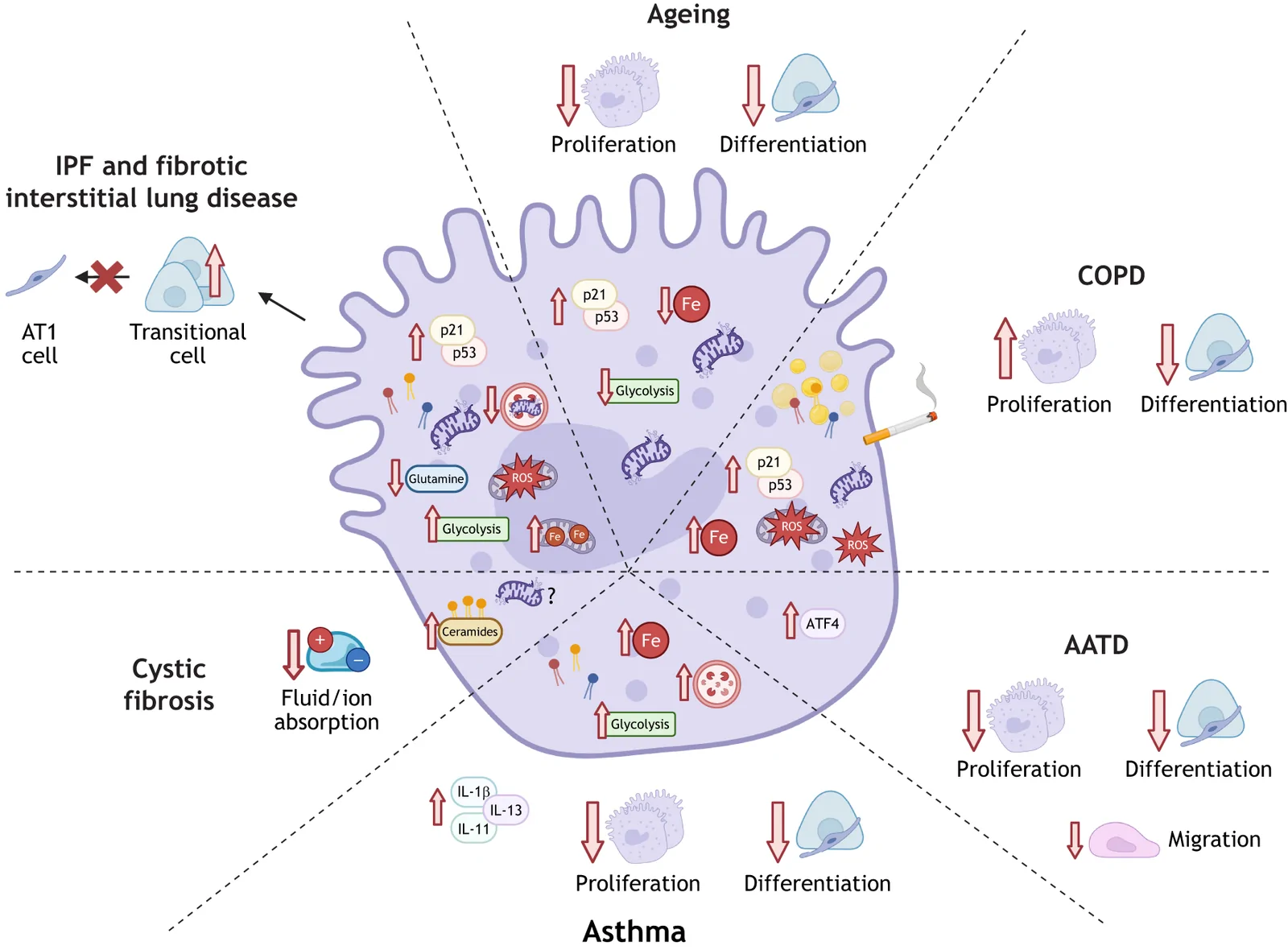
**Dysregulated AT2 cell metabolism is associated with impaired regeneration in ageing and chronic lung diseases.** Aged AT2 cells are characterised by mitochondrial dysfunction, impaired glycolytic ATP generation, functional iron insufficiency and increased senescence (indicated by increased p21 and p53), which are associated with impaired regenerative functions. Failure of AT2 cell differentiation and accumulation of transitional cells in IPF and fibrotic interstitial lung diseases is associated with reduced glutamine, dysregulated lipid metabolism, mitochondrial iron overload, impaired mitophagy, a shift toward increased glycolysis and increased senescence in AT2 cells. AT2 cells in cystic fibrosis exhibit ceramide accumulation, mitochondrial dysfunction and impaired fluid/ion absorption. Inflammatory IL-1β, IL-13 and IL-11 signalling in asthma drives the impairment of AT2 cell regenerative functions through dysregulation of glycolytic and lipid metabolism, autophagy and induction of ferroptosis. Impaired alveolar regeneration in COPD is associated with a shift toward enhanced AT2 cell proliferation and decline in differentiation, as well as increased AT2 cell senescence and ferroptosis, likely fuelled by mitochondrial dysfunction and the accumulation of intracellular iron, ROS and oxidised lipids. Impaired regeneration in AATD emphysema is associated with cellular stress responses and impaired AT2 cell migration to sites of injury. AATD, alpha-1-antitrypsin deficiency; AT1, alveolar type 1; COPD, chronic obstructive pulmonary disease; Fe, iron; IL, interleukin; IPF, idiopathic pulmonary fibrosis; ROS, reactive oxygen species. Created in BioRender by Cloonan, S. (2026). https://BioRender.com/xjhx8ze. This figure was sublicensed under CC-BY 4.0 terms.

**Table 1.** Summary of literature on dysregulated AT2 cell metabolism in chronic lung diseases

Disease	Metabolic dysregulation	Supporting literature
IPF	↑Glycolysis Mitochondrial structure, dynamics and function Impaired autophagy Mitochondrial iron overload Lipid deficiency Disrupted FAS and FAO ↓Glutamine metabolism Senescence	Newton et al., 2021; Rodríguez et al., 2025 Bueno et al., 2015; Patel et al., 2015; Chung et al., 2019; He et al., 2026 Shao et al., 2024 Chung et al., 2019; Liang et al., 2024; Yang et al., 2024; Rodríguez et al., 2025 Wang et al., 2022b Yao et al., 2021; Su et al., 2024
COPD	Dysregulated lipid metabolism ↑FAS ↑FAO? Mitochondrial damage ↓OXPHOS, ↑ROS ↑Iron, ferroptosis Senescence	Fan et al., 2023; Agudelo et al., 2020; Agarwal et al., 2014; Zhu et al., 2025; Irie et al., 2022; Tsutsumi et al., 2020 Mizumura et al., 2014; van der Toorn et al., 2007; Hara et al., 2013; Hoffmann et al., 2013; Kosmider et al., 2019 Yoshida et al., 2019; Wang and Xia, 2023; Nouws et al., 2026 Peng et al., 2024; Tsuji et al., 2006; Tsuji et al., 2004
AATD	Cellular stress and senescence ↓Migration	Abo et al., 2024 Farber et al., 2025
Asthma	Dysregulated lipid metabolism Ferroptosis	Heeley et al., 2000; Liu et al., 1997 Tang et al., 2021; Han et al., 2021; Ma et al., 2024; Zhang et al., 2024
Cystic fibrosis	Ceramide accumulation ↓Liquid absorption	Ulrich et al., 2010; Teichgräber et al., 2008 Li et al., 2012

AATD, alpha-1-antitrypsin deficiency; AT2, alveolar type 2; COPD, chronic obstructive pulmonary disease; FAO, fatty acid oxidation; FAS, fatty acid synthesis; IPF, idiopathic pulmonary fibrosis; OXPHOS, oxidative phosphorylation; ROS, reactive oxygen species.

### Metabolic dysregulation with ageing

Ageing is associated with a progressive decline in AT2 cell regenerative capacity, and age-related metabolic dysfunction likely drives this regenerative failure of AT2 cells, promoting the augmented development of chronic lung diseases with age. Single-cell analyses of aged murine lungs, together with studies of human aged lung tissue, have identified a decline in AT2 cell numbers and the emergence of inflammatory AT2 cell populations with impaired proliferative and differentiation capacity (Quan et al., 2024; Liu et al., 2024; Zhuang et al., 2025; Wang et al., 2023; de Man et al., 2026). Across aged mouse and human lung samples, AT2 cells increasingly exhibit features of cellular senescence, including irreversible cell-cycle arrest, increased oxidative stress and acquisition of a senescence-associated secretory phenotype (Quan et al., 2024; Liu et al., 2024; de Man et al., 2026), and AT2 cell senescence is sufficient to give rise to spontaneous, progressive lung fibrosis (Yao et al., 2021). In aged murine bleomycin injury models, AT2 cells exhibit reduced AMPK signalling and GlycoATP production, resulting in impaired regenerative signalling and differentiation to AT1 cells (Wang et al., 2023). However, mitochondrial dysfunction promotes cellular senescence through activation of p53 (also known as TP53) signalling via AMPK sensing of low NAD^+^ (Wiley et al., 2016), and AT2 cell senescence is associated with altered OXPHOS and enhanced oxidative lipid metabolism (Lv et al., 2025). Lastly, studies of aged murine AT2 cells have further identified functional iron insufficiency and impaired zinc metabolism, both of which suppress AT2 stem function (Zhuang et al., 2025; Liang et al., 2022). Overall, age-associated metabolic dysregulation and related AT2 cell senescence heighten the susceptibility to aberrant repair following injury, thereby promoting the development of age-related lung diseases including pulmonary fibrosis and emphysema.

### Idiopathic pulmonary fibrosis

IPF is a devastating progressive lung disease with a median survival of only 3-5 years after diagnosis (Kaunisto et al., 2019; Ley et al., 2011). It is characterised by irreversible fibrotic scarring and stiffening of the lung tissue, significantly impairing lung capacity (Jiang et al., 2025). One of the prevailing theories of IPF pathobiology suggests the emergence of a perpetual cycle of dysregulated regenerative signalling in response to repetitive microinjuries to the alveolar epithelium, driving a shift towards fibrosis rather than repair (Confalonieri et al., 2022). Consequently, AT2 cells are emerging as critical mediators of lung fibrosis, with dysfunctional repair mechanisms impairing alveolar regeneration and driving fibrotic remodelling (Parimon et al., 2020). In IPF, AT2 cells display a progressively reduced regenerative potential (Choi et al., 2025), with enriched signatures of cellular senescence (Yao et al., 2021; Su et al., 2024). Additionally, a subset of AT2 cells becomes stuck in the transitional cell state during differentiation, leading to the accumulation and persistence of aberrant transitional cells displaying gene signatures of epithelial–mesenchymal transition and senescence (Strunz et al., 2020; Kobayashi et al., 2020). Although the metabolic profile of these aberrant transitional cells remains to be extensively characterised, the metabolism of AT2 cells in IPF has been assessed.

Accumulating evidence establishes mitochondrial damage in AT2 cells as a central mechanism for IPF pathogenesis (Bueno et al., 2015; Patel et al., 2015). AT2 cells in murine models of bleomycin-induced lung injury and fibrosis and human IPF lungs show perturbed mitochondrial dynamics and display abnormal mitochondrial morphologies, indicating impaired mitochondrial homeostasis and quality control (Chung et al., 2019; Bueno et al., 2015). Dysregulated mitochondrial dynamics may impair oxidative metabolism, redox balance and organelle quality control. A recent study highlights the loss of *MPC2* in AT2 cells from patients with IPF, which is associated with altered mitochondrial respiration and impaired AT2 cell regenerative functions (He et al., 2026). Furthermore, mitochondrial iron overload in AT2 cells has been observed in murine and *in vitro* bleomycin models of IPF (Shao et al., 2024), suggesting disruption of normal mitochondrial function. Mitochondrial damage is also associated with dysregulated lipid metabolism, including disrupted FAS and FAO, exacerbating murine bleomycin-induced models of fibrosis by impairing AT2 cell regenerative functions (Chung et al., 2019; Liang et al., 2024; Yang et al., 2024). Additionally, glutamine metabolism, essential for AT2-cell mediated repair, is reduced in AT2 cells from patients with IPF and bleomycin-treated mice, potentially contributing to aberrant profibrotic responses and impaired epithelial regeneration (Wang et al., 2022b). Together, these findings indicate that multiple mitochondrial pathways are compromised in IPF.

Consistent with this decline in mitochondrial function, several studies have revealed a shift toward enhanced glycolysis (Newton et al., 2021; Rodríguez et al., 2025), likely to compensate for the loss of mitochondrial energy production. Importantly, using both IPF patient induced pluripotent stem cell-derived (iPSC-derived) AT2^I73T^ cells and mice modelling the same patient-derived variant in SFTPC (SP-CI73T), Rodríguez et al. (2025) identified deficient AMPK signalling as a pathogenic mechanism leading to the impairment of AT2 cell self-renewal and the accumulation of transitional cells (Rodríguez et al., 2025). Pathological AT2 cells that accumulate within the transitional cell state exhibited enhanced glycolytic metabolism, downregulated phospholipid and fatty acid metabolism, and disrupted mitochondrial function, including reduced FAO, as a result of a progressive decline in AMPK signalling while these cells enter the transitional state (Rodríguez et al., 2025). Because successful terminal differentiation requires a metabolic shift away from glycolysis and toward OXPHOS, these maladaptive metabolic programmes may represent a mechanism by which AT2 cells become stuck in the transitional state in IPF. Notably, these conclusions are primarily derived from murine and iPSC-derived models, and direct metabolic characterisation of transitional cells isolated from lungs of patients with IPF remains limited. However, restoration of AMPK signalling corrected these metabolic abnormalities and alleviated fibrosis development *in vivo* in mice (Rodríguez et al., 2025), suggesting that correct metabolic regulation of AT2 and transitional cells is crucial in preventing, and possibly reversing, the fibrotic lung phenotype. Further supporting a role for dysregulated AT2 cell lipid homeostasis in fibrotic lung disease, phospholipid accumulation within lamellar bodies has been implicated in Hermansky–Pudlak syndrome-associated pulmonary fibrosis, a disorder characterised by AT2 cell hyperplasia and progressive fibrotic remodelling (Kook et al., 2016; Gochuico et al., 2012).

Lastly, it has been widely established that high glycolytic metabolism, dysregulated lipid metabolism and mitochondrial dysfunction, comparable to the metabolic profile seen in AT2 cells in IPF, are linked to the development of cellular senescence (Wiley and Campisi, 2016, 2021; Kwon et al., 2019). Although the mechanisms of how dysregulated metabolism can give rise to senescent phenotypes are described in more detail elsewhere (Wiley and Campisi, 2016, 2021; Kwon et al., 2019), it is important to highlight that the similar metabolic profile in AT2 cells in IPF may promote senescence and, thus, impair alveolar regeneration.

Overall, AT2 cells demonstrate significant impairment of mitochondrial function along with maladaptive glycolytic compensation, potentially driving the emergence of senescent and aberrant transitional cells in IPF.

### Fibrotic interstitial lung diseases

Interstitial lung diseases (ILDs) comprise over 200 chronic lung disorders spanning inflammatory to fibrotic phenotypes, with IPF the most common type of fibrotic ILD (fILD), alongside others including sarcoidosis, hypersensitivity pneumonitis and autoimmune/connective tissue-related ILDs, such as rheumatoid arthritis-associated ILD and systemic sclerosis-associated ILD (Althobiani et al., 2024). Across fILDs, a unifying feature is repetitive epithelial injury leading to AT2 cell dysfunction and characterised by metabolic stress, loss of progenitor function, and acquisition of profibrotic and senescent phenotypes. This mechanism is best established in IPF, as discussed above, but similar processes are increasingly implicated across other fILDs (Wuyts et al., 2013; Pianigiani et al., 2026; Boyle et al., 2025), although direct experimental validation outside the IPF paradigm remains limited.

Common risk factors and genetic backgrounds overlap between IPF and other fILDs and converge on AT2 injury and dysfunction. Key similarities include the accumulation of transitional AT2 cell states, senescence and impaired regeneration via metabolic stress, defective autophagy/mitophagy, dysregulated regenerative pathways, such as TGF-β/Wnt signalling, and profibrotic signalling (Katzen and Beers, 2020; Cerro Chiang and Parimon, 2023; Liu et al., 2025b; Moss et al., 2022). However, some differences between subtypes exist. For example, although there is marked AT2 cell loss in IPF, AT2 cell numbers may be preserved in systemic sclerosis-associated ILD (Valenzi et al., 2019), suggesting functional or metabolic reprogramming rather than numeric depletion. AT2 cell functional decline in hypersensitivity pneumonitis is more strongly associated with chronic inflammation (Choi et al., 2020; Wang et al., 2022a), while sarcoidosis pathology involves granulomatous inflammation and hypoxia likely imposing mitochondrial and oxidative stress on AT2 cells (McGarry et al., 2018). Despite these distinct triggers, single-cell ILD datasets spanning multiple fILDs (IPF, autoimmune/connective tissue-related ILD, fibrotic hypersensitivity pneumonitis, sarcoidosis) have identified similar AT2 cell-derived transitional populations (Choi et al., 2020; Yao et al., 2021; Moss et al., 2022). This suggests that maladaptive AT2 lineage intermediates represent a common feature of fILD pathogenesis even when triggers differ. Although direct metabolic measurements are largely limited to IPF and bleomycin models, available pan-ILD single-cell RNA-sequencing datasets and pathobiology support that AT2 cell quality control failure, mitochondrial dysfunction, glycolytic reprogramming and profibrotic transitional cells are common endpoints across fILDs (Katzen and Beers, 2020; Cerro Chiang and Parimon, 2023).

### Chronic obstructive pulmonary disease

COPD is a leading cause of death worldwide and is characterised by chronic airway inflammation and emphysema (Agustí et al., 2023). Although extensive loss of alveolar epithelial cells due to exacerbated apoptosis remains a primary contributor to emphysema (Calabrese et al., 2005), impairment or loss of regenerative potential in AT2 cells is gaining more attention as a driving force of emphysema development. Emerging literature notes overall impaired AT2 cell regenerative capacity in lungs from patients with COPD, including the emergence of emphysema-associated AT2 cell populations with reduced progenitor potential (Hu et al., 2024). Studies in murine CS exposure models of COPD further suggest a shift in AT2 cell regenerative behaviour toward enhanced proliferation (Tsutsumi et al., 2020; Irie et al., 2022), accompanied by impaired differentiation into mature AT1 cells (Yu et al., 2022). Additionally, AT2 cells show characteristics of stress-induced premature senescence in both lungs from individuals with COPD and experimental murine models (Peng et al., 2024; Tsuji et al., 2006, 2004). Metabolic dysfunction in AT2 cells, particularly in response to CS exposure, a primary risk factor for COPD, is increasingly recognised as a central instigator of COPD pathogenesis.

Various literature has established altered pulmonary surfactant composition in human COPD (Agudelo et al., 2020), suggesting dysregulated lipid metabolism in AT2 cells. AT2 cells isolated from murine CS exposure models exhibit increased FASN activity after CS exposure, associated with an altered surfactant lipid profile (Fan et al., 2023; Agarwal et al., 2014) and lipid accumulation (Zhu et al., 2025). Considering the literature indicating that increased FAS impairs AT2 cell regenerative functions, this suggests a mechanism by which CS-induced lipidomic dysregulation diminishes the alveolar repair capacity. Conversely, short-term and intermittent CS exposure in mice promoted AT2 cell proliferation through enhanced FAO (Tsutsumi et al., 2020; Irie et al., 2022). This apparent discrepancy may be explained by the duration and stage of the regenerative response being examined. Acute enhancement of FAO may provide metabolic support for early AT2 cell activation and proliferation, whereas chronic CS exposure appears to result in broader lipid metabolic dysfunction that may, ultimately, impair progression through regenerative differentiation programmes compromising long-term alveolar repair.

Prolonged CS exposure further induces mitochondrial damage in human airway epithelial cells, bronchial epithelial cell models and murine CS exposure models, in which it is associated with mitochondrial fragmentation and impaired OXPHOS metabolism (Mizumura et al., 2014; van der Toorn et al., 2007; Hara et al., 2013; Hoffmann et al., 2013; Kosmider et al., 2019). Therefore, chronic CS exposure can lead to persistent metabolic dysfunction, cell stress and apoptosis. Although apoptosis and necroptosis have traditionally been considered the predominant mechanisms of epithelial cell loss in COPD, accumulating evidence indicates that ferroptosis is induced in lung epithelial cells upon CS exposure and may contribute substantially to AT2 cell depletion and regenerative failure (Yoshida et al., 2019; Wang and Xia, 2023). Mechanistically, a recent study using lung tissue from individuals with COPD and experimental models suggests that loss of the ferroptosis inhibitor nuclear protein 1 (NUPR1) in COPD lungs contributes to iron accumulation, lipid peroxidation, oxidative stress and impaired mitochondrial function in AT2 cells (Nouws et al., 2026). Increased ROS through CS exposure may also accelerate epithelial cell senescence (Hara et al., 2013; Rivas et al., 2022), impairing regeneration. It is important to note, however, that most of these studies focus on upper airway epithelial cells rather than the alveolar epithelium; therefore, their findings may not be directly applicable to AT2 cells.

Lastly, downregulation of integrin subunit alpha 3 (ITGA3), a regulator of AT2 cell proliferation through the FAK (also known as PTK2)/YAP (also known as YAP1) axis (DiGiovanni et al., 2023), caused by CS-induced ROS accumulation in murine CS exposure models, resulted in impaired AT2 cell proliferation (Liu et al., 2025a). ITGA3 has been implicated in promoting cancer growth by boosting glycolysis through *HIF1A* (Li et al., 2025), thus further linking CS-induced metabolic dysregulation to disrupted AT2 cell regenerative function. However, AT2 cell-specific mechanisms of metabolic regulation of regeneration through ITGA3 have yet to be investigated.

Collectively, these findings support the concept that chronic CS exposure drives a coordinated disruption of lipid metabolism, mitochondrial function and redox balance in AT2 cells. This metabolic disturbance promotes oxidative stress, ferroptosis-like injury and sustained impairment of AT2 cell repair functions, ultimately contributing to defective alveolar regeneration and progressive emphysematous remodelling in COPD.

### Alpha-1-antitrypsin deficiency

Alpha-1-antitrypsin (AAT; also known as SERPINA1) is a serine protease inhibitor that protects the lung parenchyma by neutralising neutrophil elastase, a protease released by activated neutrophils that degrades structural lung proteins and promotes inflammatory signalling (Strnad et al., 2020). One of the most common genetic conditions affecting the lung is AAT deficiency (AATD), in which point variations in AAT allow unopposed proteolytic activity, predisposing homozygotes to early-onset emphysema (Strnad et al., 2020). AAT was recently found to be expressed by lung AT2 cells and macrophages (Abo et al., 2024). In AATD, the pathogenic variant Z-AAT undergoes aberrant folding and polymerization, resulting in inefficient secretion and intracellular accumulation, which lead to lung injury and impaired AT2 cell regenerative capacity in mice (Magallón Serrano et al., 2026). However, it is unclear whether intracellular Z-AAT polymerisation occurs in AT2 cells as it does in hepatocytes, in which retention of misfolded Z-AAT in the hepatocyte endoplasmic reticulum disrupts cellular bioenergetics through altering mitochondrial morphology, increasing oxygen consumption and remodelling mitochondrial permeability (Yang and Kim, 2025; Abo et al., 2024; Khodayari et al., 2021). AT2 cells in AATD show increased expression of genes involved in inflammation and the unfolded protein response, along with increased ATF4 signalling (a regulator of amino acid metabolism and autophagy) and expression of cleaved caspase 3 (a trigger for apoptosis), indicating increased cellular stress (Yan and Liu, 2025; Abo et al., 2024). Moreover, AAT promotes the migration of *in vitro* AT2-like A549 cells towards sites of cellular injury and death (Farber et al., 2025). Thus, metabolic and proteostatic stress, as well as impaired migration of AAT-deficient AT2 cells to sites of injury, may contribute to insufficient alveolar repair during emphysema progression. A possible mechanism is through reduced canonical WNT/β-catenin signalling in AATD, which is associated with poor AT2 cell proliferation and early senescence, although definitive mechanisms remain to be elucidated (Magallon Serrano et al., 2025).

## Therapeutic targeting of metabolic pathways to restore AT2 cell regenerative potential

We have reviewed accumulating literature establishing metabolic regulation as a central determinant of AT2 cell regenerative capacity and suggesting that metabolic dysfunction represents a convergent mechanism underlying impaired lung repair in chronic lung diseases. Therapeutic targeting of metabolic pathways, particularly those governing glycolysis, energy sensing, mitochondrial quality control and redox balance, may offer a rational strategy to restore AT2 cell regenerative functions in disease.

A growing body of work highlights glycolytic metabolism as a key feature of AT2 cell proliferation. Recent evidence demonstrates that AT2 cells in aged and fibrotic lungs exhibit reduced AMPK activity, and impaired ATP production, resulting in defective alveolar regeneration following injury (Wang et al., 2023). Notably, restoration of AMPK–PFKFB2 signalling was sufficient to rescue post-injury alveolar repair in aged mice, underscoring the therapeutic potential for glycolysis activation in age-associated regenerative decline (Wang et al., 2023). Translational relevance is further supported by recent work using murine and human iPSC-derived AT2 cell models of IPF exhibiting deficiency of AMPK activity. Here, the authors demonstrate that AMPK agonists, such as 5-aminoimidazole-4-carboxamide ribonucleotide (AICAR) and metformin, rescued dysregulated AT2 cell metabolism and subsequent fibrogenesis (Rodríguez et al., 2025). It is important to consider, however, that broad activation of glycolysis may impair terminal AT2 cell differentiation as previously discussed. Consequently, therapeutic strategies aimed at enhancing glycolysis should be temporally controlled and selectively targeted to early regenerating cells that require increased glycolytic activity, such as proliferating and early-stage transitional AT2 cells.

Beyond glycolysis, mitochondrial fitness is increasingly recognised as a prerequisite for effective epithelial repair. Disruption of mitophagy exacerbates oxidative stress and impairs epithelial regeneration in the lung; therefore, antioxidant therapies may be beneficial in promoting lung epithelial regeneration (Li et al., 2020). Additionally, restoring aberrant AT2 cell lipid metabolism, for instance, by counteracting lipid deficiencies with exogenous lipid supplementation, can restore AT2 cell regenerative capacity in models of IPF (Liang et al., 2024). Targeting lipid metabolism may not only promote AT2 cell-mediated regeneration but also restore pulmonary surfactant homeostasis, a key dysregulation in many chronic lung diseases.

Lastly, pharmacologically targeting p53-dependent senescence in AT2 cells suppressed lung fibrogenesis (Yao et al., 2021), and the use of senolytic drugs that selectively ablate senescent cells to potentially attenuate lung fibrosis is gaining traction in the field (Lehmann et al., 2017; Justice et al., 2019; Yao et al., 2021). However, studies are limited to experimental trials in mouse models (Lehmann et al., 2017; Yao et al., 2021), while the development of senolytic drugs for use in human lung disease is only at the very earliest stages of feasibility and tolerance testing (Justice et al., 2019).

As AT2 cell metabolism in lung regeneration is an emerging field of research, studies targeting dysfunctional metabolism to restore lung regeneration are limited. However, based on the accumulating evidence that cellular metabolism directly regulates repair, targeting one or multiple branches of AT2 cell metabolism may provide promising treatment strategies for reprogramming AT2 cells toward an enhanced regenerative potential to promote lung repair. Nevertheless, implementation of therapeutic metabolites or metabolic inhibitors poses extensive challenges.

## Future directions

As evidenced, cellular metabolism is a highly complex system consisting of many interconnected pathways that are continuously and dynamically remodelled in response to cellular state and in regeneration. Several key challenges, in addition to our limited understanding of metabolite-directed regulation of AT2-cell mediated regeneration, remain to be addressed to translate these fundamental biological insights into effective therapeutic strategies.

To date, most studies have focused on individual biological pathways in isolation, often modulating them globally in ways that affect both proliferation and differentiation. However, how the timely activation or inhibition of these pathways selectively regulates these processes remains unclear. Metabolic targeting of AT2 cell regeneration will likely require precision modulation rather than global pathway activation, involving combinatorial and sequential therapies that engage distinct metabolic branches at defined stages of repair. Appropriate timing of these therapies will be key to effective repair, as a recent study suggested that metabolite supplementation exerts opposing effects when administered before or after injury (Gokey et al., 2021). Therapeutic strategies that fail to account for these temporal dynamics during repair may be ineffective or even detrimental, raising the possibility that general metabolic pathway activation can disrupt regenerative trajectories rather than enhance them. Hence, one of the most critical future directions is the need to precisely define how distinct metabolic programs are temporally coordinated with AT2 cell fate decisions during lung repair. Currently very few studies carefully characterise the transitional AT2 cell state using early- versus late-stage markers. Furthermore, the transient nature of these populations has limited their prospective isolation and direct functional interrogation, meaning that many current insights into KRT8^+^ transitional cell metabolism remain inferred from transcriptomic analyses rather than direct metabolic measurements. Future studies combining lineage tracing with time-resolved single-cell transcriptomics, proteomics and metabolomics will be essential to map temporal metabolic transitions at high resolution. Such approaches may reveal metabolic checkpoints that gate progression through transitional regenerative states and may prove more effective targets than broadly enhancing proliferation or differentiation alone.

Importantly, it also remains unclear whether metabolic pathways primarily regulate epithelial regeneration through cell-autonomous effects on AT2 cell fate or indirectly through broader effects on inflammation, stromal remodelling and tissue repair. Most studies infer regenerative mechanisms from tissue-level outcomes, making it difficult to distinguish cellular regeneration from repair of the lung. Future studies employing mosaic AT2-specific genetic targeting strategies, in which metabolically perturbed and unperturbed AT2 cells coexist within the same injured lung, could help define the cell-autonomous metabolic programmes that directly govern AT2 self-renewal, transitional state progression and differentiation.

Importantly, cellular metabolism needs to remain adaptable to simultaneous challenges such as recurrent infections, which are prevalent in individuals with COPD, for instance. While shifting cellular metabolism toward a pro-regenerative phenotype to promote lung repair, AT2 cells still need to be able to respond to and shift their metabolism accordingly in times of infection, eliminating permanent metabolic pathway alterations as therapeutic options. Because core metabolic pathways are shared across cell types, metabolism-altering therapies also need to be highly specific to the target cells to avoid impairing effector functions of other cell types, such as immune cells in the microenvironment. Advances in pulmonary drug delivery including alveolar-targeted inhaled compounds and cell-targeted metabolic modulators are needed to enhance AT2 cell specificity and limit off-target effects. Therapeutic agents utilising AT2 cell-specific uptake mechanisms or prodrugs activated by AT2 cell-specific enzymes could preferentially target AT2 cells while limiting their action on other cells. Ultimately, effective AT2-specific metabolic therapy will likely require multi-layered precision, combining targeted delivery, context-dependent activation and temporal control.

A notable limitation of the current field is its heavy reliance on murine bleomycin injury models. Although these systems have generated many of the mechanistic insights discussed throughout this Review, including links between glycolysis, OXPHOS, lipid metabolism, amino acid metabolism and AT2 cell fate, bleomycin injury incompletely recapitulates the chronic, heterogeneous and progressive nature of human lung diseases, such as IPF and COPD. Furthermore, many mechanistic studies infer metabolic function through perturbation of metabolic enzymes and pathways rather than direct manipulation of individual metabolites, making it difficult to distinguish pathway-dependent effects from metabolite-specific functions. Consequently, the extent to which metabolic programmes identified during acute alveolar repair govern regeneration in human disease remains incompletely understood. Future studies integrating human tissues, patient-derived organoids, iPSC models, spatial metabolomics, isotope tracing and longitudinal analyses of disease progression will be essential to establish the translational relevance of pharmacological modulation of metabolism and metabolite-specific mechanisms underlying alveolar regeneration.

Despite these challenges, the collective evidence reviewed here supports the concept that metabolic reprogramming is not merely a consequence of alveolar regeneration but an active regulator of AT2 cell fate and tissue repair. Emerging studies suggest that coordinated metabolic transitions govern progression from AT2 stem cells through transitional differentiation states to mature AT1 cells, and that disruption of these programmes contributes to regenerative failure in ageing and chronic lung disease. Future studies aimed at generating high-resolution temporal maps of metabolic rewiring across defined AT2-to-AT1 transitional states will be critical for establishing the precise metabolic requirements of successful alveolar regeneration. A deeper understanding of these metabolic programmes will inform the development of innovative therapies aimed at restoring lung repair and preventing irreversible tissue damage.

## References

[DMM052978C1] Abo, K. M., Merritt, C., Basil, M. C., Lin, S. M., Cantu, E., Morley, M. P., Bawa, P., Gallagher, M., Byers, D. E., Morrisey, E. E. et al. (2024). Pulmonary cellular toxicity in alpha-1 antitrypsin deficiency. *Chest* 166, 472-479. 10.1016/j.chest.2024.02.013 PMC11443245 38360172

[DMM052978C2] Agarwal, A. R., Yin, F. and Cadenas, E. (2014). Short-term cigarette smoke exposure leads to metabolic alterations in lung alveolar cells. *Am. J. Respir. Cell Mol. Biol.* 51, 284-293. 10.1165/rcmb.2013-0523OC 24625219

[DMM052978C3] Agudelo, C. W., Kumley, B. K., Area-Gomez, E., Xu, Y., Dabo, A. J., Geraghty, P., Campos, M., Foronjy, R. and Garcia-Arcos, I. (2020). Decreased surfactant lipids correlate with lung function in chronic obstructive pulmonary disease (COPD). *PLoS ONE* 15, e0228279. 10.1371/journal.pone.0228279 PMC7004328 32027677

[DMM052978C4] Agustí, A., Celli, B. R., Criner, G. J., Halpin, D., Anzueto, A., Barnes, P., Bourbeau, J., Han, M. K., Martinez, F. J., Montes de Oca, M. et al. (2023). Global initiative for chronic obstructive lung disease 2023 report: GOLD executive summary. *Eur. Respir. J.* 61, 2300239. 10.1183/13993003.00239-2023 PMC10066569 36858443

[DMM052978C5] Althobiani, M. A., Russell, A. M., Jacob, J., Ranjan, Y., Folarin, A. A., Hurst, J. R. and Porter, J. C. (2024). Interstitial lung disease: a review of classification, etiology, epidemiology, clinical diagnosis, pharmacological and non-pharmacological treatment. *Front. Med.* 11, 1296890. 10.3389/fmed.2024.1296890 PMC11063378 38698783

[DMM052978C6] Andersson, S., Bui, H., Viitanen, A., Borshagovski, D., Salminen, E., Kilpinen, S., Gebhart, A., Kuuluvainen, E., Gopalakrishnan, S., Peltokangas, N. et al. (2025). Old mitochondria regulate niche renewal via α-ketoglutarate metabolism in stem cells. *Nat. Metab.* 7, 1344-1357. 10.1038/s42255-025-01325-7 PMC12286850 40659911

[DMM052978C7] Angeles-Lopez, Q. D., Rodriguez-Lopez, J., Agudelo Garcia, P., Calyeca, J., Álvarez, D., Bueno, M., Tu, L. N., Salazar-Terreros, M., Vanegas-Avendaño, N., Krull, J. E. et al. (2025). Regulation of lung progenitor plasticity and repair by fatty acid oxidation. *JCI Insight* 10, e165837. 10.1172/jci.insight.165837 PMC11948574 39927460

[DMM052978C8] Arts, R. J., Novakovic, B., Ter Horst, R., Carvalho, A., Bekkering, S., Lachmandas, E., Rodrigues, F., Silvestre, R., Cheng, S.-C., Wang, S.-Y. et al. (2016). Glutaminolysis and fumarate accumulation integrate immunometabolic and epigenetic programs in trained immunity. *Cell Metab.* 24, 807-819. 10.1016/j.cmet.2016.10.008 PMC5742541 27866838

[DMM052978C9] Babaei-Jadidi, R., Clements, D., Wu, Y., Chen, K., Miller, S., Platé, M., Lim, K., Rue, R., Krymskaya, V. P., Chambers, R. et al. (2025). mTOR dysregulation induces IL-6 and paracrine AT2 cell senescence impeding lung repair in lymphangioleiomyomatosis. *Nat. Commun.* 16, 8996. 10.1038/s41467-025-64036-3 PMC12511327 41068078

[DMM052978C10] Bai, X., Feng, M., Kim, R. Y., Donovan, C., Anwer, A., Padula, M. P., Wang, M., Chan, Y. L., Chen, H. and Oliver, B. G. (2025). Vitamin C attenuates low-level PM2.5 exposure-induced lung inflammation and mitochondrial loss. *Environ. Int.* 206, 109929. 10.1016/j.envint.2025.109929 41252941

[DMM052978C11] Barkauskas, C. E., Cronce, M. J., Rackley, C. R., Bowie, E. J., Keene, D. R., Stripp, B. R., Randell, S. H., Noble, P. W. and Hogan, B. L. (2013). Type 2 alveolar cells are stem cells in adult lung. *J. Clin. Invest.* 123, 3025-3036. 10.1172/JCI68782 PMC3696553 23921127

[DMM052978C168] Basil, M. C., Alysandratos, K. D., Kotton, D. N. and Morrisey, E. E. (2024). Lung repair and regeneration: Advanced models and insights into human disease. *Cell Stem Cell* 31, 439-454. 10.1016/j.stem.2024.02.009 PMC11070171 38492572

[DMM052978C12] Besnard, V., Wert, S. E., Stahlman, M. T., Postle, A. D., Xu, Y., Ikegami, M. and Whitsett, J. A. (2009). Deletion of Scap in alveolar type II cells influences lung lipid homeostasis and identifies a compensatory role for pulmonary lipofibroblasts. *J. Biol. Chem.* 284, 4018-4030. 10.1074/jbc.M805388200 PMC2635058 19074148

[DMM052978C13] Boyle, N., Miller, J., Quinn, S., Maguire, J., Fabre, A., Morrisroe, K., Murphy, D. J. and McCarthy, C. (2025). Systemic autoimmune rheumatic diseases-associated interstitial lung disease: a pulmonologist's perspective. *Breathe* 21, 240171. 10.1183/20734735.0171-2024 PMC12171859 40529316

[DMM052978C14] Brown, L. A. S., Harris, F. L. and Jones, D. P. (1997). Ascorbate deficiency and oxidative stress in the alveolar type II cell. *Am. J. Physiol. Lung Cell. Mol. Physiol.* 273, L782-L788. 10.1152/ajplung.1997.273.4.L782 9357853

[DMM052978C15] Bueno, M., Lai, Y. C., Romero, Y., Brands, J., St Croix, C. M., Kamga, C., Corey, C., Herazo-Maya, J. D., Sembrat, J., Lee, J. S. et al. (2015). PINK1 deficiency impairs mitochondrial homeostasis and promotes lung fibrosis. *J. Clin. Invest.* 125, 521-538. 10.1172/JCI74942 PMC4319413 25562319

[DMM052978C16] Calabrese, F., Giacometti, C., Beghe, B., Rea, F., Loy, M., Zuin, R., Marulli, G., Baraldo, S., Saetta, M. and Valente, M. (2005). Marked alveolar apoptosis/proliferation imbalance in end-stage emphysema. *Respir. Res.* 6, 14. 10.1186/1465-9921-6-14 PMC549521 15705190

[DMM052978C17] Carey, B. W., Finley, L. W., Cross, J. R., Allis, C. D. and Thompson, C. B. (2015). Intracellular α-ketoglutarate maintains the pluripotency of embryonic stem cells. *Nature* 518, 413-416. 10.1038/nature13981 PMC4336218 25487152

[DMM052978C18] Cerro Chiang, G. and Parimon, T. (2023). Understanding interstitial lung diseases associated with connective tissue disease (CTD-ILD): genetics, cellular pathophysiology, and biologic drivers. *Int. J. Mol. Sci.* 24, 2405. 10.3390/ijms24032405 PMC9917355 36768729

[DMM052978C19] Chaves-Perez, A., Millman, S. E., Janaki-Raman, S., Ho, Y.-J., Hinterleitner, C., Barthet, V. J. A., Morris, J. P., Barriga, F. M., Reyes, J., Kyaw, A. et al. (2025). Metabolic adaptations direct cell fate during tissue regeneration. *Nature* 643, 468-477. 10.1038/s41586-025-09097-6 PMC12240837 40500453

[DMM052978C20] Chen, C., Liu, Y., Liu, R., Ikenoue, T., Guan, K. L., Liu, Y. and Zheng, P. (2008). TSC-mTOR maintains quiescence and function of hematopoietic stem cells by repressing mitochondrial biogenesis and reactive oxygen species. *J. Exp. Med.* 205, 2397-2408. 10.1084/jem.20081297 PMC2556783 18809716

[DMM052978C21] Cheng, H., Feng, D., Li, X., Gao, L., Tang, S., Liu, W., Wu, X., Yue, S., Li, C. and Luo, Z. (2021). Iron deposition-induced ferroptosis in alveolar type II cells promotes the development of pulmonary fibrosis. *Biochim. Biophys. Acta Mol. Basis Dis.* 1867, 166204. 10.1016/j.bbadis.2021.166204 34175430

[DMM052978C22] Cho, Y. M., Kwon, S., Pak, Y. K., Seol, H. W., Choi, Y. M., Park, D. J., Park, K. S. and Lee, H. K. (2006). Dynamic changes in mitochondrial biogenesis and antioxidant enzymes during the spontaneous differentiation of human embryonic stem cells. *Biochem. Biophys. Res. Commun.* 348, 1472-1478. 10.1016/j.bbrc.2006.08.020 16920071

[DMM052978C23] Choi, J., Park, J. E., Tsagkogeorga, G., Yanagita, M., Koo, B. K., Han, N. and Lee, J. H. (2020). Inflammatory signals induce AT2 cell-derived damage-associated transient progenitors that mediate alveolar regeneration. *Cell Stem Cell* 27, 366-382.e7. 10.1016/j.stem.2020.06.020 PMC7487779 32750316

[DMM052978C24] Choi, H. K., Bang, G., Shin, J. H., Shin, M. H., Woo, A., Kim, S. Y., Lee, S. H., Kim, E. Y., Shim, H. S., Suh, Y. J. et al. (2025). Regenerative capacity of alveolar type 2 cells is proportionally reduced following disease progression in idiopathic pulmonary fibrosis-derived organoid cultures. *Tuberc. Respir Dis.* 88, 130-137. 10.4046/trd.2024.0094 PMC11704724 39343426

[DMM052978C25] Chung, K.-P., Hsu, C.-L., Fan, L.-C., Huang, Z., Bhatia, D., Chen, Y.-J., Hisata, S., Cho, S. J., Nakahira, K., Imamura, M. et al. (2019). Mitofusins regulate lipid metabolism to mediate the development of lung fibrosis. *Nat. Commun.* 10, 3390. 10.1038/s41467-019-11327-1 PMC6662701 31358769

[DMM052978C26] Chung, K.-P., Cheng, C.-N., Chen, Y.-J., Hsu, C.-L., Huang, Y.-L., Hsieh, M.-S., Kuo, H.-C., Lin, Y.-T., Juan, Y.-H., Nakahira, K. et al. (2024). Alveolar epithelial cells mitigate neutrophilic inflammation in lung injury through regulating mitochondrial fatty acid oxidation. *Nat. Commun.* 15, 7241. 10.1038/s41467-024-51683-1 PMC11341863 39174557

[DMM052978C27] Confalonieri, P., Volpe, M. C., Jacob, J., Maiocchi, S., Salton, F., Ruaro, B., Confalonieri, M. and Braga, L. (2022). Regeneration or repair? The role of alveolar epithelial cells in the pathogenesis of idiopathic pulmonary fibrosis (IPF). *Cells* 11, 2095. 10.3390/cells11132095 PMC9266271 35805179

[DMM052978C28] Covarrubias, A. J., Perrone, R., Grozio, A. and Verdin, E. (2021). NAD^+^ metabolism and its roles in cellular processes during ageing. *Nat. Rev. Mol. Cell Biol.* 22, 119-141. 10.1038/s41580-020-00313-x PMC7963035 33353981

[DMM052978C29] Crotta, S., Villa, M., Major, J., Finsterbusch, K., Llorian, M., Carmeliet, P., Buescher, J. and Wack, A. (2023). Repair of airway epithelia requires metabolic rewiring towards fatty acid oxidation. *Nat. Commun.* 14, 721. 10.1038/s41467-023-36352-z PMC9925445 36781848

[DMM052978C30] Cui, Y., Robertson, J., Maharaj, S., Waldhauser, L., Niu, J., Wang, J., Farkas, L., Kolb, M. and Gauldie, J. (2011). Oxidative stress contributes to the induction and persistence of TGF-β1 induced pulmonary fibrosis. *Int. J. Biochem. Cell Biol.* 43, 1122-1133. 10.1016/j.biocel.2011.04.005 21514399

[DMM052978C31] Cui, H., Xie, N., Banerjee, S., Ge, J., Guo, S. and Liu, G. (2019). Impairment of fatty acid oxidation in alveolar epithelial cells mediates acute lung injury. *Am. J. Respir. Cell Mol. Biol.* 60, 167-178. 10.1165/rcmb.2018-0152OC PMC6376408 30183330

[DMM052978C32] Das, D. K. (1980). Fatty acid synthesis in fetal lung. *Biochem. Biophys. Res. Commun.* 92, 867-875. 10.1016/0006-291X(80)90783-4 7362611

[DMM052978C33] de Man, R., McDonough, J. E., Adams, T. S., Nikola, F., Rangel, R., Anderson, S., Manning, E. P., Cala Garcia, J., Moss, B., Waich, A. et al. (2026). Single-cell atlas of human lung aging identifies cell type dyssynchrony and increased transcriptional entropy. *Nat. Commun.* 17, 2095. 10.1038/s41467-026-68810-9 PMC12953888 41571679

[DMM052978C34] Desai, T. J., Brownfield, D. G. and Krasnow, M. A. (2014). Alveolar progenitor and stem cells in lung development, renewal and cancer. *Nature* 507, 190-194. 10.1038/nature12930 PMC4013278 24499815

[DMM052978C35] Digiovanni, G. T., Han, W., Sherrill, T. P., Taylor, C. J., Nichols, D. S., Geis, N. M., Singha, U. K., Calvi, C. L., McCall, A. S., Dixon, M. M. et al. (2023). Epithelial Yap/Taz are required for functional alveolar regeneration following acute lung injury. *JCI Insight* 8, e173374. 10.1172/jci.insight.173374 PMC10629815 37676731

[DMM052978C36] Fan, L.-C., McConn, K., Plataki, M., Kenny, S., Williams, N. C., Kim, K., Quirke, J. A., Chen, Y., Sauler, M., Möbius, M. E. et al. (2023). Alveolar type II epithelial cell FASN maintains lipid homeostasis in experimental COPD. *JCI Insight* 8, e163403. 10.1172/jci.insight.163403 PMC10543729 37606038

[DMM052978C37] Fang, T., Yang, J., Liu, L., Xiao, H. and Wei, X. (2021). Nicotinamide mononucleotide ameliorates senescence in alveolar epithelial cells. *MedComm* 2, 279-287. 10.1002/mco2.62 PMC8491199 34766147

[DMM052978C38] Farber, I., Wated, M., Schuster, R., Sheffer, L., Anav, Y., Ogen-Shtern, N., Tsitrina, A., Tacruri, D., Bunin, A., Nagar, N. B. et al. (2025). Alpha-1 antitrypsin promotes re-epithelialization by regulating inflammation and migration. *Front. Immunol.* 16, 1586039. 10.3389/fimmu.2025.1586039 PMC12162329 40519926

[DMM052978C39] Fujimoto, S., Hirano, Y., Kawajiri, K., Yoshida, M., Inukai, S., Nishioka, S., Matsubayashi, S., Watanabe, N., Ito, S., Minagawa, S. et al. (2026). Extracellular vesicle cargo from lipofibroblasts mitigates chronic obstructive pulmonary disease by alveolar type 2 cell restoration. *Am. J. Respir. Crit. Care. Med.* 212, 1237-1252. 10.1093/ajrccm/aamag025 PMC13255518 41738201

[DMM052978C40] Gao, R.-W., Kong, X.-Y., Zhu, X.-X., Zhu, G.-Q., Ma, J.-S. and Liu, X.-X. (2015). Retinoic acid promotes primary fetal alveolar epithelial type II cell proliferation and differentiation to alveolar epithelial type I cells. *In Vitro Cell. Dev. Biol. Anim.* 51, 479-487. 10.1007/s11626-014-9850-2 25515249

[DMM052978C41] Gochuico, B. R., Huizing, M., Golas, G. A., Scher, C. D., Tsokos, M., Denver, S. D., Frei-Jones, M. J. and Gahl, W. A. (2012). Interstitial lung disease and pulmonary fibrosis in Hermansky-Pudlak syndrome type 2, an adaptor protein-3 complex disease. *Mol. Med.* 18, 56-64. 10.2119/molmed.2011.00198 PMC3269640 22009278

[DMM052978C42] Gokey, J. J., Snowball, J., Green, J., Waltamath, M., Spinney, J. J., Black, K. E., Hariri, L. P., Xu, Y. and Perl, A. K. (2021). Pretreatment of aged mice with retinoic acid supports alveolar regeneration via upregulation of reciprocal PDGFA signalling. *Thorax* 76, 456-467. 10.1136/thoraxjnl-2020-214986 PMC8070612 33479039

[DMM052978C43] Gui, Y. S., Wang, L., Tian, X., Li, X., Ma, A., Zhou, W., Zeng, N., Zhang, J., Cai, B., Zhang, H. et al. (2015). mTOR overactivation and compromised autophagy in the pathogenesis of pulmonary fibrosis. *PLoS ONE* 10, e0138625. 10.1371/journal.pone.0138625 PMC4575195 26382847

[DMM052978C169] Han, F., Li, S., Yang, Y. and Bai, Z. (2021). Interleukin-6 promotes ferroptosis in bronchial epithelial cells by inducing reactive oxygen species-dependent lipid peroxidation and disrupting iron homeostasis. *Bioengineered* 12, 5279-5288. 10.1080/21655979.2021.1964158 PMC8806540 34402724

[DMM052978C44] Han, S., Lee, M., Shin, Y., Giovanni, R., Chakrabarty, R. P., Herrerias, M. M., Dada, L. A., Flozak, A. S., Reyfman, P. A., Khuder, B. et al. (2023). Mitochondrial integrated stress response controls lung epithelial cell fate. *Nature* 620, 890-897. 10.1038/s41586-023-06423-8 PMC10447247 37558881

[DMM052978C45] Hara, H., Araya, J., Ito, S., Kobayashi, K., Takasaka, N., Yoshii, Y., Wakui, H., Kojima, J., Shimizu, K., Numata, T. et al. (2013). Mitochondrial fragmentation in cigarette smoke-induced bronchial epithelial cell senescence. *Am. J. Physiol. Lung Cell. Mol. Physiol.* 305, L737-L746. 10.1152/ajplung.00146.2013 24056969

[DMM052978C46] Hardie, D. G., Ross, F. A. and Hawley, S. A. (2012). AMPK: a nutrient and energy sensor that maintains energy homeostasis. *Nat. Rev. Mol. Cell Biol.* 13, 251-262. 10.1038/nrm3311 PMC5726489 22436748

[DMM052978C47] Harijith, A., Basa, P., Ha, A., Thomas, J., Jafri, A., Fu, P., MacFarlane, P. M., Raffay, T. M., Natarajan, V. and Sudhadevi, T. (2022). NOX4 mediates epithelial cell death in hyperoxic acute lung injury through mitochondrial reactive oxygen species. *Front. Pharmacol.* 13, 880878. 10.3389/fphar.2022.880878 PMC9160661 35662702

[DMM052978C48] He, Z., Li, X., Miao, X., Xu, G., Wang, S., Li, L., Wu, Q. and Chen, H. (2026). Mitochondrial pyruvate metabolism controls alveolar stem cell fate and inflammatory balance. *Inflammation* 49, 101. 10.1007/s10753-026-02470-1 PMC12979318 41776090

[DMM052978C170] Heeley, E. L., Hohlfeld, J. M., Krug, N. and Postle, A. D. (2000). Phospholipid molecular species of bronchoalveolar lavage fluid after local allergen challenge in asthma. *Am. J. Physiol. Lung Cell Mol. Physiol.* 278, L305-11. 10.1152/ajplung.2000.278.2.L305 10666114

[DMM052978C49] Ho, T. T., Warr, M. R., Adelman, E. R., Lansinger, O. M., Flach, J., Verovskaya, E. V., Figueroa, M. E. and Passegué, E. (2017). Autophagy maintains the metabolism and function of young and old stem cells. *Nature* 543, 205-210. 10.1038/nature21388 PMC5344718 28241143

[DMM052978C50] Hoffmann, R. F., Zarrintan, S., Brandenburg, S. M., Kol, A., de Bruin, H. G., Jafari, S., Dijk, F., Kalicharan, D., Kelders, M., Gosker, H. R. et al. (2013). Prolonged cigarette smoke exposure alters mitochondrial structure and function in airway epithelial cells. *Respir. Res.* 14, 97. 10.1186/1465-9921-14-97 PMC3852998 24088173

[DMM052978C51] Hu, Y., Hu, Q., Ansari, M., Riemondy, K., Pineda, R., Sembrat, J., Leme, A. S., Ngo, K., Morgenthaler, O., Ha, K. et al. (2024). Airway-derived emphysema-specific alveolar type II cells exhibit impaired regenerative potential in COPD. *Eur. Respir. J.* 64, 2302071. 10.1183/13993003.02071-2023 PMC11618816 39147413

[DMM052978C52] Irie, H., Ozaki, M., Chubachi, S., Hegab, A. E., Tsutsumi, A., Kameyama, N., Sakurai, K., Nakayama, S., Kagawa, S., Wada, S. et al. (2022). Short-term intermittent cigarette smoke exposure enhances alveolar type 2 cell stemness via fatty acid oxidation. *Respir. Res.* 23, 41. 10.1186/s12931-022-01948-4 PMC8889685 35236337

[DMM052978C53] Ito, K., Hirao, A., Arai, F., Matsuoka, S., Takubo, K., Hamaguchi, I., Nomiyama, K., Hosokawa, K., Sakurada, K., Nakagata, N. et al. (2004). Regulation of oxidative stress by ATM is required for self-renewal of haematopoietic stem cells. *Nature* 431, 997-1002. 10.1038/nature02989 15496926

[DMM052978C54] Jang, Y. Y. and Sharkis, S. J. (2007). A low level of reactive oxygen species selects for primitive hematopoietic stem cells that may reside in the low-oxygenic niche. *Blood* 110, 3056-3063. 10.1182/blood-2007-05-087759 PMC2018677 17595331

[DMM052978C55] Jiang, M., Bu, W., Wang, X., Ruan, J., Shi, W., Yu, S., Huang, L., Xue, P., Tang, J., Zhao, X. et al. (2025). Pulmonary fibrosis: from mechanisms to therapies. *J. Transl. Med.* 23, 515. 10.1186/s12967-025-06514-2 PMC12063347 40340941

[DMM052978C56] Justice, J. N., Nambiar, A. M., Tchkonia, T., LeBrasseur, N. K., Pascual, R., Hashmi, S. K., Prata, L., Masternak, M. M., Kritchevsky, S. B., Musi, N. et al. (2019). Senolytics in idiopathic pulmonary fibrosis: results from a first-in-human, open-label, pilot study. *EBioMedicine* 40, 554-563. 10.1016/j.ebiom.2018.12.052 PMC6412088 30616998

[DMM052978C57] Katzen, J. and Beers, M. F. (2020). Contributions of alveolar epithelial cell quality control to pulmonary fibrosis. *J. Clin. Invest.* 130, 5088-5099. 10.1172/JCI139519 PMC7524463 32870817

[DMM052978C58] Kaunisto, J., Salomaa, E. R., Hodgson, U., Kaarteenaho, R., Kankaanranta, H., Koli, K., Vahlberg, T. and Myllärniemi, M. (2019). Demographics and survival of patients with idiopathic pulmonary fibrosis in the FinnishIPF registry. *ERJ Open Res.* 5, 00170-2018. 10.1183/23120541.00170-2018 PMC6612605 31304177

[DMM052978C59] Khacho, M., Clark, A., Svoboda, D. S., Azzi, J., MacLaurin, J. G., Meghaizel, C., Sesaki, H., Lagace, D. C., Germain, M., Harper, M. E. et al. (2016). Mitochondrial dynamics impacts stem cell identity and fate decisions by regulating a nuclear transcriptional program. *Cell Stem Cell* 19, 232-247. 10.1016/j.stem.2016.04.015 27237737

[DMM052978C60] Khodayari, N., Wang, R. L., Oshins, R., Lu, Y., Millett, M., Aranyos, A. M., Mostofizadeh, S., Scindia, Y., Flagg, T. O. and Brantly, M. (2021). The mechanism of mitochondrial injury in Alpha-1 antitrypsin deficiency mediated liver disease. *Int. J. Mol. Sci.* 22, 13255. 10.3390/ijms222413255 PMC8704552 34948056

[DMM052978C61] Kobayashi, Y., Tata, A., Konkimalla, A., Katsura, H., Lee, R. F., Ou, J., Banovich, N. E., Kropski, J. A. and Tata, P. R. (2020). Persistence of a regeneration-associated, transitional alveolar epithelial cell state in pulmonary fibrosis. *Nat. Cell Biol.* 22, 934-946. 10.1038/s41556-020-0542-8 PMC7461628 32661339

[DMM052978C62] Kook, S., Wang, P., Young, L. R., Schwake, M., Saftig, P., Weng, X., Meng, Y., Neculai, D., Marks, M. S., Gonzales, L. et al. (2016). Impaired lysosomal integral membrane protein 2-dependent peroxiredoxin 6 delivery to lamellar bodies accounts for altered alveolar phospholipid content in adaptor protein-3-deficient pearl mice. *J. Biol. Chem.* 291, 8414-8427. 10.1074/jbc.M116.720201 PMC4861416 26907692

[DMM052978C63] Kosmider, B., Lin, C. R., Karim, L., Tomar, D., Vlasenko, L., Marchetti, N., Bolla, S., Madesh, M., Criner, G. J. and Bahmed, K. (2019). Mitochondrial dysfunction in human primary alveolar type II cells in emphysema. *EBioMedicine* 46, 305-316. 10.1016/j.ebiom.2019.07.063 PMC6711885 31383554

[DMM052978C64] Kwak, S., Song, C. L., Cho, Y. S., Choi, I., Byun, J.-E., Jung, H. and Lee, J. (2024). Txnip regulates the Oct4-mediated pluripotency circuitry via metabolic changes upon differentiation. *Cell. Mol. Life Sci.* 81, 142. 10.1007/s00018-024-05161-y PMC10940461 38485770

[DMM052978C65] Kwon, S. M., Hong, S. M., Lee, Y.-K., Min, S. and Yoon, G. (2019). Metabolic features and regulation in cell senescence. *BMB Rep.* 52, 5-12. 10.5483/BMBRep.2019.52.1.291 PMC6386228 30526768

[DMM052978C66] Lalioti, M.-E., Romero-Mulero, M. C., Karabacz, N., Mess, J., Demollin, H., Rettkowski, J., Schuldes, K., Mitterer, M., Wadle, C., Shoumariyeh, K. et al. (2025). Differentiation, ageing and leukaemia alter the metabolic profile of human bone marrow haematopoietic stem and progenitor cells. *Nat. Cell Biol.* 27, 1367-1380. 10.1038/s41556-025-01709-7 PMC12339397 40664811

[DMM052978C67] Lamprecht, J., ABU Sanad, S., Kusumbe, A. P. and Habib, S. J. (2026). The WntNAD^+^ axis in cancer, aging, and tissue regeneration. *Trends Cell Biol.* 36, 154-174. 10.1016/j.tcb.2025.07.006 40885694

[DMM052978C68] Laplante, M. and Sabatini, D. M. (2009). mTOR signaling at a glance. *J. Cell Sci.* 122, 3589-3594. 10.1242/jcs.051011 PMC2758797 19812304

[DMM052978C69] Lehmann, M., Korfei, M., Mutze, K., Klee, S., Skronska-Wasek, W., Alsafadi, H. N., Ota, C., Costa, R., Schiller, H. B., Lindner, M. et al. (2017). Senolytic drugs target alveolar epithelial cell function and attenuate experimental lung fibrosis ex vivo. *Eur. Respir. J.* 50, 1602367. 10.1183/13993003.02367-2016 PMC5593348 28775044

[DMM052978C70] Ley, B., Collard, H. R. and King, T. E. Jr. (2011). Clinical course and prediction of survival in idiopathic pulmonary fibrosis. *Am. J. Respir. Crit. Care. Med.* 183, 431-440. 10.1164/rccm.201006-0894CI 20935110

[DMM052978C171] Li, X., Comellas, A. P., Karp, P. H., Ernst, S. E., Moninger, T. O., Gansemer, N. D., Taft, P. J., Pezzulo, A. A., Rector, M. V., Rossen, N. et al. (2012). CFTR is required for maximal transepithelial liquid transport in pig alveolar epithelia. *Am. J. Physiol. Lung Cell. Mol. Physiol.* 303, L152-60. 10.1152/ajplung.00116.2012 PMC3404631 22637155

[DMM052978C71] Li, X., Wu, J., Sun, X., Wu, Q., Li, Y., Li, K., Zhang, Q., Li, Y., Abel, E. D. and Chen, H. (2020). Autophagy reprograms alveolar progenitor cell metabolism in response to lung injury. *Stem Cell Rep.* 14, 420-432. 10.1016/j.stemcr.2020.01.008 PMC7066233 32059792

[DMM052978C72] Li, S., Liberti, D., Zhou, S., Ying, Y., Kong, J., Basil, M. C., Cardenas-Diaz, F. L., Shiraishi, K., Morley, M. P. and Morrisey, E. E. (2023). DOT1L regulates lung developmental epithelial cell fate and adult alveolar stem cell differentiation after acute injury. *Stem Cell Rep.* 18, 1841-1853. 10.1016/j.stemcr.2023.07.006 PMC10545485 37595582

[DMM052978C73] Li, R., Ji, Q., Fu, S., Gu, J., Liu, D., Wang, L., Yuan, X., Wen, Y., Dai, C. and Li, H. (2025). ITGA3 promotes pancreatic cancer progression through HIF1α- and c-Myc-driven glycolysis in a collagen I-dependent autocrine manner. *Cancer Gene Ther.* 32, 240-253. 10.1038/s41417-024-00864-7 39690180

[DMM052978C74] Liang, J., Huang, G., Liu, X., Taghavifar, F., Liu, N., Wang, Y., Deng, N., Yao, C., Xie, T., Kulur, V. et al. (2022). The ZIP8/SIRT1 axis regulates alveolar progenitor cell renewal in aging and idiopathic pulmonary fibrosis. *J. Clin. Invest.* 132, e157338. 10.1172/JCI157338 PMC9151700 35389887

[DMM052978C75] Liang, J., Huang, G., Liu, X., Zhang, X., Rabata, A., Liu, N., Fang, K., Taghavifar, F., Dai, K., Kulur, V. et al. (2024). Lipid deficiency contributes to impaired alveolar progenitor cell function in aging and idiopathic pulmonary fibrosis. *Am. J. Respir. Cell Mol. Biol.* 71, 242-253. 10.1165/rcmb.2023-0290OC PMC11299087 38657143

[DMM052978C76] Lin, C. R., Bahmed, K. and Kosmider, B. (2022). Impaired alveolar re-epithelialization in pulmonary emphysema. *Cells* 11, 2055. 10.3390/cells11132055 PMC9265977 35805139

[DMM052978C172] Liu, M., Wang, L., Holm, B. A. and Enhorning, G. (1997). Dysfunction of guinea-pig pulmonary surfactant and type II pneumocytes after repetitive challenge with aerosolized ovalbumin. *Clin. Exp. Allergy* 27, 802-807. 10.1046/j.1365-2222.1997.420885.x 9249273

[DMM052978C77] Liu, J., Qin, X., Pan, D., Zhang, B. and Jin, F. (2019). Amino acid-mediated metabolism: a new power to influence properties of stem cells. *Stem Cells Int.* 2019, 6919463. 10.1155/2019/6919463 PMC6915148 31885621

[DMM052978C78] Liu, X., Zhang, X., Yao, C., Liang, J., Noble, P. W. and Jiang, D. (2024). Transcriptomics analysis identifies the decline in the alveolar type II stem cell niche in aged human lungs. *Am. J. Respir. Cell Mol. Biol.* 71, 229-241. 10.1165/rcmb.2023-0363OC PMC11299088 38635761

[DMM052978C79] Liu, L., Zhong, S., Zhou, T., Wu, Y., Hu, W., Wang, X., Ren, Y., Wang, K., Lin, X. and Zhang, J. (2025a). Dysregulated ITGA3/FAK/YAP axis mediates impaired alveolar type II epithelial cells function in COPD. *J. Adv. Res.* 86, 639-653. 10.2991/978-94-6463-932-2 PMC13453853 41270956

[DMM052978C80] Liu, S., Xi, Q., Li, X. and Liu, H. (2025b). Mitochondrial dysfunction and alveolar type II epithelial cell senescence: The destroyer and rescuer of idiopathic pulmonary fibrosis. *Front. Cell Dev. Biol.* 13, 1535601. 10.3389/fcell.2025.1535601 PMC11994736 40230412

[DMM052978C81] Lottes, R. G., Newton, D. A., Spyropoulos, D. D. and Baatz, J. E. (2014). Alveolar type II cells maintain bioenergetic homeostasis in hypoxia through metabolic and molecular adaptation. *Am. J. Physiol. Lung Cell. Mol. Physiol.* 306, L947-L955. 10.1152/ajplung.00298.2013 PMC4025059 24682450

[DMM052978C82] Ludin, A., Gur-Cohen, S., Golan, K., Kaufmann, K. B., Itkin, T., Medaglia, C., Lu, X.-J., Ledergor, G., Kollet, O. and Lapidot, T. (2014). Reactive oxygen species regulate hematopoietic stem cell self-renewal, migration and development, as well as their bone marrow microenvironment. *Antioxid Redox Signal.* 21, 1605-1619. 10.1089/ars.2014.5941 PMC4175025 24762207

[DMM052978C83] Lunt, S. Y. and Vander Heiden, M. G. (2011). Aerobic glycolysis: meeting the metabolic requirements of cell proliferation. *Annu. Rev. Cell Dev. Biol.* 27, 441-464. 10.1146/annurev-cellbio-092910-154237 21985671

[DMM052978C84] Luo, Y., Li, L., Zou, P., Wang, J., Shao, L., Zhou, D. and Liu, L. (2014). Rapamycin enhances long-term hematopoietic reconstitution of ex vivo expanded mouse hematopoietic stem cells by inhibiting senescence. *Transplantation* 97, 20-29. 10.1097/TP.0b013e3182a7fcf8 PMC3877183 24092377

[DMM052978C85] Lv, X., Chen, Q., Zhou, Z., Wu, W., Yu, X. and Zeng, Y. (2025). αCGRP deficiency aggravates pulmonary fibrosis by promoting senescence in alveolar type 2 cells. *Genes Immun.* 26, 589-598. 10.1038/s41435-025-00361-3 PMC12705422 41023452

[DMM052978C86] Lyon, P., Strippoli, V., Fang, B. and Cimmino, L. (2020). B vitamins and one-carbon metabolism: implications in human health and disease. *Nutrients* 12, 2867. 10.3390/nu12092867 PMC7551072 32961717

[DMM052978C173] Ma, L., He, Y., Xie, H., Wang, J., Chen, J., Song, S., Zhang, L., Li, L., Lai, H., Liu, Y. et al. (2024). Ferroptotic alveolar epithelial type II cells drive TH2 and TH17 mixed asthma triggered by birch pollen allergen Bet v 1. *Cell Death Discov.* 10, 96. 10.1038/s41420-024-01861-3 PMC10891108 38395974

[DMM052978C87] Magallon Serrano, M., Mohammad, N., Lee, J., Aranyos, A., Bowers, W., Griffin, A., Patel, S., Lascano, J., Flagg-Dowie, T., Brantly, M. L. et al. (2025). Impaired wingless and int-1(WNT) signaling in type 2 alveolar epithelial cells drives senescence and inefficient alveolar repair in alpha-1 antitrypsin deficient lungs. *Am. J. Respir. Crit. Care. Med.* 211, A3061. 10.1164/ajrccm.2025.211.Abstracts.A3061

[DMM052978C174] Magallón Serrano, M., Khodayari, N., Bowers, W., Manning, E. P., Liu, X., Lee, J., Flagg, T. O., Oshins, R., Griffin, A., Patel, S. et al. (2026). Polymerized Z-α-1 antitrypsin leads to lung injury in a murine model of emphysema. *JCI Insight* 11, e199029. 10.1172/jci.insight.199029 PMC13463610 42579796

[DMM052978C88] Maniscalco, W. M., Finkelstein, J. N. and Parkhurst, A. B. (1989). Effects of exogenous fatty acids and inhibition of de novo fatty acid synthesis on disaturated phosphatidylcholine production by fetal lung cells and adult type II cells. *Exp. Lung Res.* 15, 473-489. 10.3109/01902148909087872 2743953

[DMM052978C89] Maryanovich, M., Zaltsman, Y., Ruggiero, A., Goldman, A., Shachnai, L., Zaidman, S. L., Porat, Z., Golan, K., Lapidot, T. and Gross, A. (2015). An MTCH2 pathway repressing mitochondria metabolism regulates haematopoietic stem cell fate. *Nat. Commun.* 6, 7901. 10.1038/ncomms8901 26219591

[DMM052978C90] Massaro, G. D., Gail, D. B. and Massaro, D. (1975). Lung oxygen consumption and mitochondria of alveolar epithelial and endothelial cells. *J. Appl. Physiol.* 38, 588-592. 10.1152/jappl.1975.38.4.588 1141087

[DMM052978C91] Maus, M., López-Polo, V., Mateo, L., Lafarga, M., Aguilera, M., De Lama, E., Meyer, K., Sola, A., Lopez-Martinez, C., López-Alonso, I. et al. (2023). Iron accumulation drives fibrosis, senescence and the senescence-associated secretory phenotype. *Nat. Metab.* 5, 2111-2130. 10.1038/s42255-023-00928-2 PMC10730403 38097808

[DMM052978C92] McGarry, T., Biniecka, M., Veale, D. and Fearon, U. (2018). Hypoxia, oxidative stress and inflammation. *Free Radic. Biol. Med.* 125, 15-24. 10.1016/j.freeradbiomed.2018.03.042 29601945

[DMM052978C93] Meng, D., Frank, A. R. and Jewell, J. L. (2018). mTOR signaling in stem and progenitor cells. *Development* 145, dev152595. 10.1242/dev.152595 PMC5825873 29311260

[DMM052978C94] Messier, E. M., Day, B. J., Bahmed, K., Kleeberger, S. R., Tuder, R. M., Bowler, R. P., Chu, H. W., Mason, R. J. and Kosmider, B. (2013). N-acetylcysteine protects murine alveolar type II cells from cigarette smoke injury in a nuclear erythroid 2-related factor-2-independent manner. *Am. J. Respir. Cell Mol. Biol.* 48, 559-567. 10.1165/rcmb.2012-0295OC PMC3707381 23492188

[DMM052978C95] Mishra, P., Carelli, V., Manfredi, G. and Chan, D. C. (2014). Proteolytic cleavage of Opa1 stimulates mitochondrial inner membrane fusion and couples fusion to oxidative phosphorylation. *Cell Metab.* 19, 630-641. 10.1016/j.cmet.2014.03.011 PMC4018240 24703695

[DMM052978C96] Mizumura, K., Cloonan, S. M., Nakahira, K., Bhashyam, A. R., Cervo, M., Kitada, T., Glass, K., Owen, C. A., Mahmood, A., Washko, G. R. et al. (2014). Mitophagy-dependent necroptosis contributes to the pathogenesis of COPD. *J. Clin. Invest.* 124, 3987-4003. 10.1172/JCI74985 PMC4151233 25083992

[DMM052978C97] Mohrin, M., Widjaja, A., Liu, Y., Luo, H. and Chen, D. (2018). The mitochondrial unfolded protein response is activated upon hematopoietic stem cell exit from quiescence. *Aging Cell* 17, e12756. 10.1111/acel.12756 PMC5946069 29575576

[DMM052978C98] Moss, B. J., Ryter, S. W. and Rosas, I. O. (2022). Pathogenic mechanisms underlying idiopathic pulmonary fibrosis. *Annu. Rev. Pathol.* 17, 515-546. 10.1146/annurev-pathol-042320-030240 34813355

[DMM052978C99] Navas, L. E. and Carnero, A. (2021). NAD^+^ metabolism, stemness, the immune response, and cancer. *Signal Transduct. Target. Ther.* 6, 2. 10.1038/s41392-020-00354-w PMC7775471 33384409

[DMM052978C100] Netea, M. G., Joosten, L. A., Latz, E., Mills, K. H., Natoli, G., Stunnenberg, H. G., O'neill, L. A. and Xavier, R. J. (2016). Trained immunity: a program of innate immune memory in health and disease. *Science* 352, aaf1098. 10.1126/science.aaf1098 PMC5087274 27102489

[DMM052978C101] Newton, D. A., Lottes, R. G., Ryan, R. M., Spyropoulos, D. D. and Baatz, J. E. (2021). Dysfunctional lactate metabolism in human alveolar type II cells from idiopathic pulmonary fibrosis lung explant tissue. *Respir. Res.* 22, 278. 10.1186/s12931-021-01866-x PMC8554831 34711218

[DMM052978C102] Nouws, J., Siller, S. S., Yang, T., Kim, S.-J., Kim, S.-H., Santofimia-Castaño, P., Iovanna, J. L., Herzog, E. L., McDonough, J. E. and Sauler, M. (2026). The ferroptosis inhibitor NUPR1 coordinates the mitochondrial response to oxidative stress and cell metabolism during COPD pathogenesis in the lung. *Am. J. Physiol. Lung Cell. Mol. Physiol.* 10.1152/ajplung.00098.2026 PMC13729395 42623300

[DMM052978C103] Oburoglu, L., Tardito, S., Fritz, V., de Barros, S. C., Merida, P., Craveiro, M., Mamede, J., Cretenet, G., Mongellaz, C., An, X. et al. (2014). Glucose and glutamine metabolism regulate human hematopoietic stem cell lineage specification. *Cell Stem Cell* 15, 169-184. 10.1016/j.stem.2014.06.002 24953180

[DMM052978C104] Papa, L., Djedaini, M. and Hoffman, R. (2019). Mitochondrial role in stemness and differentiation of hematopoietic stem cells. *Stem Cells Int.* 2019, 4067162. 10.1155/2019/4067162 PMC6381553 30881461

[DMM052978C105] Parimon, T., Yao, C., Stripp, B. R., Noble, P. W. and Chen, P. (2020). Alveolar epithelial type II cells as drivers of lung fibrosis in idiopathic pulmonary fibrosis. *Int. J. Mol. Sci.* 21, 2269. 10.3390/ijms21072269 PMC7177323 32218238

[DMM052978C106] Patel, A. S., Song, J. W., Chu, S. G., Mizumura, K., Osorio, J. C., Shi, Y., El-Chemaly, S., Lee, C. G., Rosas, I. O., Elias, J. A. et al. (2015). Epithelial cell mitochondrial dysfunction and PINK1 are induced by transforming growth factor-beta1 in pulmonary fibrosis. *PLoS ONE* 10, e0121246. 10.1371/journal.pone.0121246 PMC4364993 25785991

[DMM052978C107] Patterson, C. E., Davis, K. S. and Rhoades, R. A. (1988). Regulation of fetal lung disaturated phosphatidylcholine synthesis by de novo palmitate supply. *Biochim. Biophys. Acta* 958, 60-69. 10.1016/0005-2760(88)90246-9 3334868

[DMM052978C108] Paul, M. K., Bisht, B., Darmawan, D. O., Chiou, R., Ha, V. L., Wallace, W. D., Chon, A. T., Hegab, A. E., Grogan, T., Elashoff, D. A. et al. (2014). Dynamic changes in intracellular ROS levels regulate airway basal stem cell homeostasis through Nrf2-dependent Notch signaling. *Cell Stem Cell* 15, 199-214. 10.1016/j.stem.2014.05.009 PMC4127166 24953182

[DMM052978C109] Payne, D. K. and Wellikof, A. (2012). *Alveolar Structure and Function*. San Rafael, Morgan & Claypool Life Sciences.

[DMM052978C110] Peng, K., Yao, Y. X., Lu, X., Wang, W. J., Zhang, Y. H., Zhao, H., Wang, H., Xu, D. X. and Tan, Z. X. (2024). Mitochondrial dysfunction-associated alveolar epithelial senescence is involved in CdCl_2_-induced COPD-like lung injury. *J. Hazard. Mater.* 476, 135103. 10.1016/j.jhazmat.2024.135103 38972203

[DMM052978C111] Pfeiffer, T., Schuster, S. and Bonhoeffer, S. (2001). Cooperation and competition in the evolution of ATP-producing pathways. *Science* 292, 504-507. 10.1126/science.1058079 11283355

[DMM052978C112] Pianigiani, T., Dilroba, A., Gambini, C., Hernandez-Gonzalez, F., D'alessandro, M., Bergantini, L., Sellares, J., Bargagli, E. and Cameli, P. (2026). Beyond inflammation: alarmins as critical drivers of pulmonary fibrosis. *ERJ Open Res.* 12, 00747-2025. 10.1183/23120541.00747-2025 PMC12969694 41809871

[DMM052978C113] Prieto, J., León, M., Ponsoda, X., Sendra, R., Bort, R., Ferrer-Lorente, R., Raya, A., López-García, C. and Torres, J. (2016). Early ERK1/2 activation promotes DRP1-dependent mitochondrial fission necessary for cell reprogramming. *Nat. Commun.* 7, 11124. 10.1038/ncomms11124 PMC4821885 27030341

[DMM052978C114] Quan, R., Shi, C., Fang, B., Sun, Y., Qu, T., Wang, X., Wang, R., Zhang, Y., Ren, F. and Li, Y. (2024). Age-dependent inflammatory microenvironment mediates alveolar regeneration. *Int. J. Mol. Sci.* 25, 3476. 10.3390/ijms25063476 PMC10970842 38542450

[DMM052978C115] Read, A. D., Bentley, R. E., Archer, S. L. and Dunham-Snary, K. J. (2021). Mitochondrial iron-sulfur clusters: structure, function, and an emerging role in vascular biology. *Redox Biol.* 47, 102164. 10.1016/j.redox.2021.102164 PMC8577454 34656823

[DMM052978C116] Ren, R., Ocampo, A., Liu, G. H. and IZPISUA Belmonte, J. C. (2017). Regulation of stem cell aging by metabolism and epigenetics. *Cell Metab.* 26, 460-474. 10.1016/j.cmet.2017.07.019 28826795

[DMM052978C117] Rindler, T. N., Stockman, C. A., Filuta, A. L., Brown, K. M., Snowball, J. M., Zhou, W., Veldhuizen, R., Zink, E. M., Dautel, S. E., Clair, G. et al. (2017). Alveolar injury and regeneration following deletion of ABCA3. *JCI Insight* 2, e97381. 10.1172/jci.insight.97381 PMC5752264 29263307

[DMM052978C118] Rivas, M., Gupta, G., Costanzo, L., Ahmed, H., Wyman, A. E. and Geraghty, P. (2022). Senescence: pathogenic driver in chronic obstructive pulmonary disease. *Medicina* 58, 817. 10.3390/medicina58060817 PMC9228143 35744080

[DMM052978C119] Rodríguez, L. R., Alysandratos, K. D., Katzen, J., Murthy, A., Roque Barboza, W., Tomer, Y., Bui, S., Acín-Pérez, R., Petcherski, A., Minakin, K. et al. (2025). Impaired AMPK control of alveolar epithelial cell metabolism promotes pulmonary fibrosis. *JCI Insight* 10, e182578. 10.1172/jci.insight.182578 PMC12333953 40591409

[DMM052978C120] Rooney, S. A., Young, S. L. and Mendelson, C. R. (1994). Molecular and cellular processing of lung surfactant. *FASEB J.* 8, 957-967. 10.1096/fasebj.8.12.8088461 8088461

[DMM052978C121] Salisbury, T. B. and Arthur, S. (2018). The regulation and function of the L-type amino acid transporter 1 (LAT1) in cancer. *Int. J. Mol. Sci.* 19, 2373. 10.3390/ijms19082373 PMC6121554 30103560

[DMM052978C122] Sanches Santos Rizzo Zuttion, M., Moore, S. K. L., Chen, P., Beppu, A. K. and Hook, J. L. (2022). New insights into the alveolar epithelium as a driver of acute respiratory distress syndrome. *Biomolecules* 12, 1273. 10.3390/biom12091273 PMC9496395 36139112

[DMM052978C123] Sappington, D. R., Siegel, E. R., Hiatt, G., Desai, A., Penney, R. B., Jamshidi-Parsian, A., Griffin, R. J. and Boysen, G. (2016). Glutamine drives glutathione synthesis and contributes to radiation sensitivity of A549 and H460 lung cancer cell lines. *Biochim. Biophys. Acta* 1860, 836-843. 10.1016/j.bbagen.2016.01.021 PMC4768472 26825773

[DMM052978C124] Schultz, C. J., Torres, E., Londos, C. and Torday, J. S. (2002). Role of adipocyte differentiation-related protein in surfactant phospholipid synthesis by type II cells. *Am. J. Physiol. Lung Cell. Mol. Physiol.* 283, L288-L296. 10.1152/ajplung.00204.2001 12114189

[DMM052978C125] Semenza, G. L. (2012). Hypoxia-inducible factors in physiology and medicine. *Cell* 148, 399-408. 10.1016/j.cell.2012.01.021 PMC3437543 22304911

[DMM052978C126] Shao, M., Cheng, H., Li, X., Qiu, Y., Zhang, Y., Chang, Y., Fu, J., Shen, M., Xu, X., Feng, D. et al. (2024). Abnormal mitochondrial iron metabolism damages alveolar type II epithelial cells involved in bleomycin-induced pulmonary fibrosis. *Theranostics* 14, 2687-2705. 10.7150/thno.94072 PMC11103499 38773980

[DMM052978C127] Shen, M., Luo, Z. and Zhou, Y. (2022). Regeneration-associated transitional state cells in pulmonary fibrosis. *Int. J. Mol. Sci.* 23, 6757. 10.3390/ijms23126757 PMC9223485 35743199

[DMM052978C128] Shiraki, N., Shiraki, Y., Tsuyama, T., Obata, F., Miura, M., Nagae, G., Aburatani, H., Kume, K., Endo, F. and Kume, S. (2014). Methionine metabolism regulates maintenance and differentiation of human pluripotent stem cells. *Cell Metab.* 19, 780-794. 10.1016/j.cmet.2014.03.017 24746804

[DMM052978C129] Shyh-Chang, N., Locasale, J. W., Lyssiotis, C. A., Zheng, Y., Teo, R. Y., Ratanasirintrawoot, S., Zhang, J., Onder, T., Unternaehrer, J. J., Zhu, H. et al. (2013). Influence of threonine metabolism on S-adenosylmethionine and histone methylation. *Science* 339, 222-226. 10.1126/science.1226603 PMC3652341 23118012

[DMM052978C130] Stolk, J., Stockley, R. A., Stoel, B. C., Cooper, B. G., Piitulainen, E., Seersholm, N., Chapman, K. R., Burdon, J. G., Decramer, M., Abboud, R. T. et al. (2012). Randomised controlled trial for emphysema with a selective agonist of the γ-type retinoic acid receptor. *Eur. Respir. J.* 40, 306-312. 10.1183/09031936.00161911 22282548

[DMM052978C131] Strnad, P., McElvaney, N. G. and Lomas, D. A. (2020). Alpha_1_-antitrypsin deficiency. *N. Engl. J. Med.* 382, 1443-1455. 10.1056/NEJMra1910234 32268028

[DMM052978C132] Strunz, M., Simon, L. M., Ansari, M., Kathiriya, J. J., Angelidis, I., Mayr, C. H., Tsidiridis, G., Lange, M., Mattner, L. F., Yee, M. et al. (2020). Alveolar regeneration through a Krt8+ transitional stem cell state that persists in human lung fibrosis. *Nat. Commun.* 11, 3559. 10.1038/s41467-020-17358-3 PMC7366678 32678092

[DMM052978C133] Su, W., Guo, Y., Wang, Q., Ma, L., Zhang, Q., Zhang, Y., Geng, Y., Jin, T., Guo, J., Yang, R. et al. (2024). YAP1 inhibits the senescence of alveolar epithelial cells by targeting Prdx3 to alleviate pulmonary fibrosis. *Exp. Mol. Med.* 56, 1643-1654. 10.1038/s12276-024-01277-0 PMC11297023 38945958

[DMM052978C134] Sun, T., Yu, H., Zhang, D., Li, D. and Fu, J. (2024). Activated DRP1 promotes mitochondrial fission and induces glycolysis in ATII cells under hyperoxia. *Respir. Res.* 25, 443. 10.1186/s12931-024-03083-8 PMC11670517 39725939

[DMM052978C175] Tang, W., Dong, M., Teng, F., Cui, J., Zhu, X., Wang, W., Wuniqiemu, T., Qin, J., Yi, L., Wang, S. et al. (2021). Environmental allergens house dust mite-induced asthma is associated with ferroptosis in the lungs. *Exp. Ther. Med.* 22, 1483. 10.3892/etm.2021.10918 PMC8576623 34765024

[DMM052978C176] Teichgräber, V., Ulrich, M., Endlich, N., Riethmüller, J., Wilker, B., De Oliveira-Munding, C. C., van Heeckeren, A. M., Barr, M. L., von Kürthy, G., Schmid, K. W. et al. (2008). Ceramide accumulation mediates inflammation, cell death and infection susceptibility in cystic fibrosis. *Nat. Med.* 14, 382-391. 10.1038/nm1748 18376404

[DMM052978C135] Tohyama, S., Fujita, J., Hishiki, T., Matsuura, T., Hattori, F., Ohno, R., Kanazawa, H., Seki, T., Nakajima, K., Kishino, Y. et al. (2016). Glutamine oxidation is indispensable for survival of human pluripotent stem cells. *Cell Metab.* 23, 663-674. 10.1016/j.cmet.2016.03.001 27050306

[DMM052978C136] Toyama, E. Q., Herzig, S., Courchet, J., Lewis, T. L. Jr, Losón, O. C., Hellberg, K., Young, N. P., Chen, H., Polleux, F., Chan, D. C. and et al. (2016). Metabolism. AMP-activated protein kinase mediates mitochondrial fission in response to energy stress. *Science* 351, 275-281. 10.1126/science.aab4138 PMC4852862 26816379

[DMM052978C137] Tsuji, T., Aoshiba, K. and Nagai, A. (2004). Cigarette smoke induces senescence in alveolar epithelial cells. *Am. J. Respir. Cell Mol. Biol.* 31, 643-649. 10.1165/rcmb.2003-0290OC 15333326

[DMM052978C138] Tsuji, T., Aoshiba, K. and Nagai, A. (2006). Alveolar cell senescence in patients with pulmonary emphysema. *Am. J. Respir. Crit. Care. Med.* 174, 886-893. 10.1164/rccm.200509-1374OC 16888288

[DMM052978C139] Tsutsumi, A., Ozaki, M., Chubachi, S., Irie, H., Sato, M., Kameyama, N., Sasaki, M., Ishii, M., Hegab, A. E., Betsuyaku, T. et al. (2020). Exposure to cigarette smoke enhances the stemness of alveolar type 2 cells. *Am. J. Respir. Cell Mol. Biol.* 63, 293-305. 10.1165/rcmb.2019-0188OC 32338993

[DMM052978C177] Ulrich, M., Worlitzsch, D., Viglio, S., Siegmann, N., Iadarola, P., Shute, J. K., Geiser, M., Pier, G. B., Friedel, G., Barr, M. L. et al. (2010). Alveolar inflammation in cystic fibrosis. *J. Cyst. Fibros.* 9, 217-227. 10.1016/j.jcf.2010.03.001 PMC2883667 20347403

[DMM052978C140] Valenzi, E., Bulik, M., Tabib, T., Morse, C., Sembrat, J., TREJO Bittar, H., Rojas, M. and Lafyatis, R. (2019). Single-cell analysis reveals fibroblast heterogeneity and myofibroblasts in systemic sclerosis-associated interstitial lung disease. *Ann. Rheum. Dis.* 78, 1379-1387. 10.1136/annrheumdis-2018-214865 PMC7255436 31405848

[DMM052978C141] van der Toorn, M., Slebos, D. J., de Bruin, H. G., Leuvenink, H. G., Bakker, S. J., Gans, R. O., Koëter, G. H., van Oosterhout, A. J. and Kauffman, H. F. (2007). Cigarette smoke-induced blockade of the mitochondrial respiratory chain switches lung epithelial cell apoptosis into necrosis. *Am. J. Physiol. Lung Cell. Mol. Physiol.* 292, L1211-L1218. 10.1152/ajplung.00291.2006 17209140

[DMM052978C142] Wang, Z. N. and Tang, X. X. (2020). New perspectives on the aberrant alveolar repair of idiopathic pulmonary fibrosis. *Front. Cell Dev. Biol.* 8, 580026. 10.3389/fcell.2020.580026 PMC7561442 33117807

[DMM052978C143] Wang, Y. and Xia, S. (2023). Relationship between ACSL4-mediated ferroptosis and chronic obstructive pulmonary disease. *Int. J. Chron. Obstruct. Pulmon. Dis.* 18, 99-111. 10.2147/COPD.S391129 PMC9930680 36817367

[DMM052978C144] Wang, C. Y., Jenkitkasemwong, S., Duarte, S., Sparkman, B. K., Shawki, A., Mackenzie, B. and Knutson, M. D. (2012). ZIP8 is an iron and zinc transporter whose cell-surface expression is up-regulated by cellular iron loading. *J. Biol. Chem.* 287, 34032-34043. 10.1074/jbc.M112.367284 PMC3464513 22898811

[DMM052978C145] Wang, J., Zhang, L., Luo, L., He, P., Xiong, A., Jiang, M., Liu, Y., Liu, S., Ran, Q., Wu, D. et al. (2022a). Characterizing cellular heterogeneity in fibrotic hypersensitivity pneumonitis by single-cell transcriptional analysis. *Cell Death Discov.* 8, 38. 10.1038/s41420-022-00831-x PMC8795750 35091537

[DMM052978C146] Wang, S., Li, X., Ma, Q., Wang, Q., Wu, J., Yu, H., Li, K., Li, Y., Wang, J., Zhang, Q. et al. (2022b). Glutamine Metabolism Is Required for Alveolar Regeneration during Lung Injury. *Biomolecules* 12, 728. 10.3390/biom12050728 PMC9138637 35625656

[DMM052978C147] Wang, Z., Wei, D., Bin, E., Li, J., Jiang, K., Lv, T., Mao, X., Wang, F., Dai, H. and Tang, N. (2023). Enhanced glycolysis-mediated energy production in alveolar stem cells is required for alveolar regeneration. *Cell Stem Cell* 30, 1028-1042.e7. 10.1016/j.stem.2023.07.007 37541209

[DMM052978C148] Wang, Y., Wang, L., Ma, S., Cheng, L. and Yu, G. (2024). Repair and regeneration of the alveolar epithelium in lung injury. *FASEB J.* 38, e23612. 10.1096/fj.202400088R 38648494

[DMM052978C149] Wang, M., Liu, C., Li, Z., Wang, Y., Li, Y. and Pei, F. (2026). Cuproptosis of bronchial epithelial cells triggers airway remodeling in chronic asthma. *Sci. Rep.* 16, 4621. 10.1038/s41598-025-34574-3 PMC12868725 41530300

[DMM052978C150] Wheaton, W. W. and Chandel, N. S. (2011). Hypoxia. 2. Hypoxia regulates cellular metabolism. *Am. J. Physiol. Cell Physiol.* 300, C385-C393. 10.1152/ajpcell.00485.2010 PMC3063979 21123733

[DMM052978C151] Wiley, C. D. and Campisi, J. (2016). From ancient pathways to aging cells-connecting metabolism and cellular senescence. *Cell Metab.* 23, 1013-1021. 10.1016/j.cmet.2016.05.010 PMC4911819 27304503

[DMM052978C152] Wiley, C. D. and Campisi, J. (2021). The metabolic roots of senescence: mechanisms and opportunities for intervention. *Nat. Metab.* 3, 1290-1301. 10.1038/s42255-021-00483-8 PMC8889622 34663974

[DMM052978C153] Wiley, C. D., Velarde, M. C., Lecot, P., Liu, S., Sarnoski, E. A., Freund, A., Shirakawa, K., Lim, H. W., Davis, S. S., Ramanathan, A. et al. (2016). Mitochondrial dysfunction induces senescence with a distinct secretory phenotype. *Cell Metab.* 23, 303-314. 10.1016/j.cmet.2015.11.011 PMC4749409 26686024

[DMM052978C154] Wu, P., Chen, J., Liu, L., Wang, P., Lu, T., Shen, S., Cao, Y., Sui, Y., Liu, R., Huan, X. et al. (2026). Mitophagy promotes lung repair and regeneration by restoring epithelial metabolic fitness. *Nat. Commun.* 17, 5170. 10.1038/s41467-026-71728-x PMC13249882 41980941

[DMM052978C155] Wuyts, W. A., Agostini, C., Antoniou, K. M., Bouros, D., Chambers, R. C., Cottin, V., Egan, J. J., Lambrecht, B. N., Lories, R., Parfrey, H. et al. (2013). The pathogenesis of pulmonary fibrosis: a moving target. *Eur. Respir. J.* 41, 1207-1218. 10.1183/09031936.00073012 23100500

[DMM052978C156] Yan, X. and Liu, C. (2025). The ATF4-glutamine axis: a central node in cancer metabolism, stress adaptation, and therapeutic targeting. *Cell Death Discov.* 11, 390. 10.1038/s41420-025-02683-7 PMC12365302 40830338

[DMM052978C157] Yang, S.-R. and Kim, H.-R. (2025). Next-generation regenerative therapies for alpha-1 antitrypsin deficiency: molecular pathogenesis to clinical translation. *Int. J. Mol. Sci.* 26, 8504. 10.3390/ijms26178504 PMC12429386 40943425

[DMM052978C158] Yang, J., Pan, X., Xu, M., Li, Y., Liang, C., Liu, L., Li, Z., Wang, L. and Yu, G. (2024). Downregulation of HMGCS2 mediated AECIIs lipid metabolic alteration promotes pulmonary fibrosis by activating fibroblasts. *Respir. Res.* 25, 176. 10.1186/s12931-024-02816-z PMC11040761 38658970

[DMM052978C159] Yao, C., Guan, X., Carraro, G., Parimon, T., Liu, X., Huang, G., Mulay, A., Soukiasian, H. J., David, G., Weigt, S. S. et al. (2021). Senescence of alveolar type 2 cells drives progressive pulmonary fibrosis. *Am. J. Respir. Crit. Care. Med.* 203, 707-717. 10.1164/rccm.202004-1274OC PMC7958503 32991815

[DMM052978C160] Yee, M., McDavid, A. N., Cohen, E. D., Huyck, H. L., Poole, C., Altman, B. J., Maniscalco, W. M., Deutsch, G. H., Pryhuber, G. S. and O'Reilly, M. A. (2022). Neonatal hyperoxia activates activating transcription factor 4 to stimulate folate metabolism and alveolar epithelial type 2 cell proliferation. *Am. J. Respir. Cell Mol. Biol.* 66, 402-414. 10.1165/rcmb.2021-0363OC PMC8990118 35045271

[DMM052978C161] Yoshida, M., Minagawa, S., Araya, J., Sakamoto, T., Hara, H., Tsubouchi, K., Hosaka, Y., Ichikawa, A., Saito, N., Kadota, T. et al. (2019). Involvement of cigarette smoke-induced epithelial cell ferroptosis in COPD pathogenesis. *Nat. Commun.* 10, 3145. 10.1038/s41467-019-10991-7 PMC6637122 31316058

[DMM052978C162] Yu, H., Lin, Y., Zhong, Y., Guo, X., Lin, Y., Yang, S., Liu, J., Xie, X., Sun, Y., Wang, D. et al. (2022). Impaired AT2 to AT1 cell transition in PM2.5-induced mouse model of chronic obstructive pulmonary disease. *Respir. Res.* 23, 70. 10.1186/s12931-022-01996-w PMC8957194 35337337

[DMM052978C163] Zeng, J., Liu, X., Bi, J., Mo, C., Zhang, H., Chai, Y., Lin, Y., Li, S., Liu, Z., Yao, M. et al. (2025). Metabolic control of stem cell function: insights into amino acid-driven mechanisms. *Stem Cell Rev. Rep.* 21, 2565-2577. 10.1007/s12015-025-10967-9 40911162

[DMM052978C164] Zhang, C. (2014). Essential functions of iron-requiring proteins in DNA replication, repair and cell cycle control. *Protein Cell* 5, 750-760. 10.1007/s13238-014-0083-7 PMC4180463 25000876

[DMM052978C165] Zhang, X., Hu, X., Zhang, Y., Liu, B., Pan, H., Liu, Z., Yao, Z., Zhu, Q., Wu, C. and Shen, T. (2023). Impaired autophagy-accelerated senescence of alveolar type II epithelial cells drives pulmonary fibrosis induced by single-walled carbon nanotubes. *J. Nanobiotechnol.* 21, 69. 10.1186/s12951-023-01821-6 PMC9970859 36849924

[DMM052978C178] Zhang, Z., He, Y., Liu, H., Liu, Y., Wu, T., Li, R., Wang, Y. and Ma, W. (2024). NLRP3 regulates ferroptosis via the JAK2/STAT3 pathway in asthma inflammation: Insights from in vivo and in vitro studies. *Int. Immunopharmacol.* 143, 113416. 10.1016/j.intimp.2024.113416 39426227

[DMM052978C166] Zhu, Y., Gamare, S., Polverino, F., Owen, C. A., Somanath, P. R., Wang, X. and Zhang, D. (2025). CYP1B1 mediates cigarette smoke-induced lipid accumulation in alveolar type 2 cells. *FASEB J.* 39, e71062. 10.1096/fj.202501439RR PMC12448152 40971194

[DMM052978C167] Zhuang, X., Wang, Q., Joost, S., Ferrena, A., Humphreys, D. T., Li, Z., Blum, M., Krause, K., Ding, S., Landais, Y. et al. (2025). Ageing limits stemness and tumorigenesis by reprogramming iron homeostasis. *Nature* 637, 184-194. 10.1038/s41586-024-08285-0 39633048

